# A Systematic Review on the Characteristics, Treatments and Outcomes of the Patients with Primary Spinal Glioblastomas or Gliosarcomas Reported in Literature until March 2015

**DOI:** 10.1371/journal.pone.0148312

**Published:** 2016-02-09

**Authors:** Stefanie Beyer, André O. von Bueren, Gunther Klautke, Matthias Guckenberger, Rolf-Dieter Kortmann, Sophie Pietschmann, Klaus Müller

**Affiliations:** 1 Center for Dentistry and Oral Medicine, University Medical Center Leipzig, Leipzig, Saxony, Germany; 2 Department of Pediatrics and Adolescent Medicine, Division of Pediatric Hematology and Oncology, University Medical Center Goettingen, Goettingen, Lower Saxony, Germany; 3 Department of Radiation Oncology, Hospital Chemnitz, Chemnitz, Germany; 4 Department of Radiation Oncology, University Hospital Zurich, Zurich, Switzerland; 5 Department of Radiation-Oncology, University Medical Center Leipzig, Leipzig, Saxony, Germany; NIH, UNITED STATES

## Abstract

Our aim was to determine the characteristics, treatments and outcomes of patients with primary spinal glioblastomas (GB) or gliosarcomas (GS) reported in literature until March 2015. PubMed and Web of Science were searched for peer-reviewed articles pertaining to cases of glioblastomas / gliosarcomas with primary spinal origin, using predefined search terms. Furthermore we performed hand searches tracking the references from the selected papers. Eighty-two articles published between 1938 and March 2015 were eligible. They reported on 157 patients. Median age at diagnosis was 22 years. The proportion of patients who received adjuvant chemo- or radiotherapy clearly increased from the time before 1980 until present. Median overall survival from diagnosis was 8.0 ± 0.9 months. On univariate analysis age influenced overall survival, whereas tumor location, gender and the extent of initial resection did not. Outcomes did not differ between children (< 18 years) and adults. However, the patients who were treated after 1980 achieved longer survival times than the patients treated before. On multivariable analysis only age (< 60 years) and the time period of treatment (≥ 1980) were confirmed as positive independent prognostic factors. In conclusion, primary spinal GB / GS mainly affect younger patients and are associated with a dismal prognosis. However, most likely due to the increasing use of adjuvant treatment, modest therapeutic progress has been achieved over recent decades. The characteristics and treatments of primary spinal glioblastomas should be entered into a central registry in order to gain more information about the ideal treatment approach in the future.

## Introduction

In adults, glioblastoma (GB) is the most frequent primary intracranial neoplasm accounting for more than 50% of all cerebral tumors [1]. In contrast, pediatric GB are rare. Data on childhood malignancies from the European Union show that only 3% of all children with central nervous system (CNS) tumors are affected by this disease [2]. Both -in adults and children- spinal origin of glioblastoma is rarely seen and accordingly, the literature on the topic is mainly restricted to single case reports or small case series. It is noteworthy that there are crucial differences between adult and pediatric malignant gliomas in terms of tumor biology and clinical course, implying that the results from adult clinical trials may not be extrapolated to children and vice versa [3]. Beyond their dismal prognosis, little is known about the clinical characteristics of primary spinal glioblastomas / gliosarcomas and their optimal treatment. By means of a systematic review referring to 157 cases of primary spinal GB / GS which were published in literature until March 2015, this paper updates and summarizes the existing knowledge on this rare disease.

## Materials and Methods

### Aim of the study

The primary objective of this study was to assess clinical characteristics, treatments and outcomes of GB / GS patients with primary spinal tumor location. Our aim was to include all cases reported in literature until March 2015. The secondary objective was to evaluate potential prognostic factors for survival.

### Search strategy and selection criteria

#### Identification

In a first step we performed PubMed and Web of Science searches with predefined search terms (for details see S1 Search Methods). We restricted our search to articles written in German, French, Spanish, Italian or English, but did not use any time limitations. In total, the search engines delivered 1509 hits, which were imported into a reference management software (endnote.com X6.0.1). After removal of duplicates, the number of hits was reduced to 1211.

#### Screening

Titles and abstracts were reviewed by two authors (SB and KM). The minimum requirement for further consideration of a case was the diagnosis of a primary spinal GB / GS, as well as information on the patient´s treatment and the course of disease.

Application of these basic inclusion criteria excluded 1126 publications after title screening and nine after abstract screening. Hand searches tracking the references from the 76 remaining articles revealed 21 further publications, which were added to the pool of papers meriting closer investigation (n = 97).

#### Eligibility

**Fig 1:**
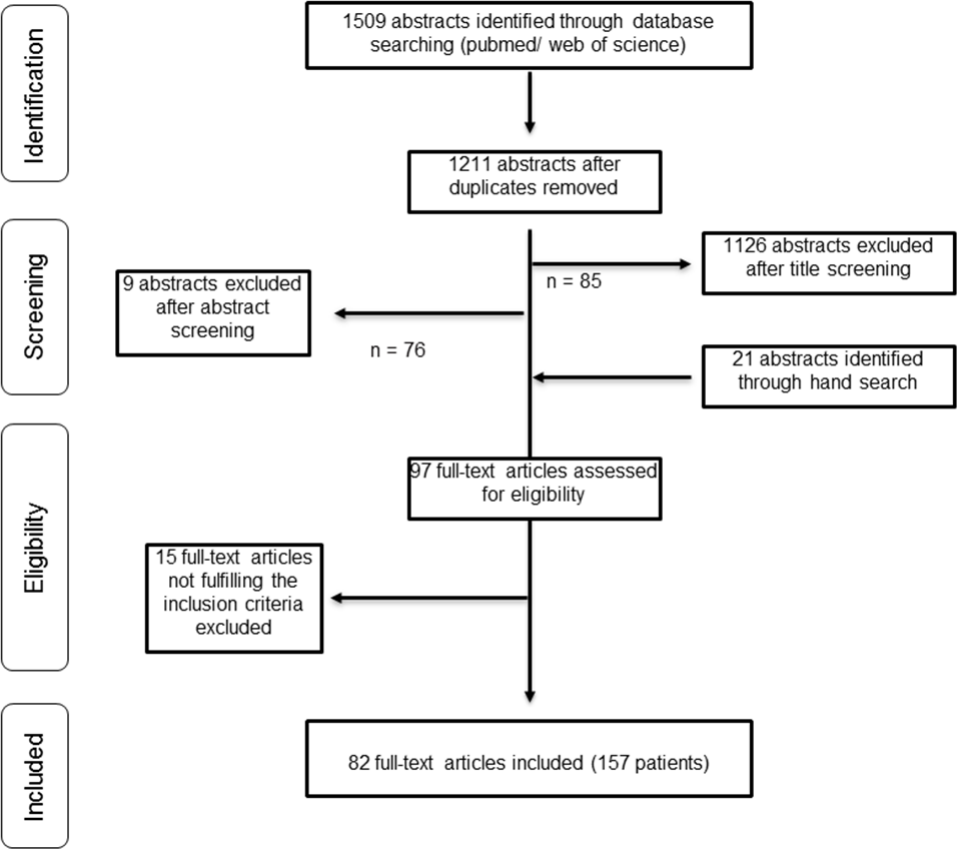
Procedure of publication retrieval and in- and exclusion of cases displayed in a PRISMA (Preferred Reporting Items for Systematic Reviews and Meta-Analyses) flow chart (4).

In total, we (SB and KM) evaluated 97 full-text articles for eligibility. Disagreements were resolved through discussion and consensus with a third author (SP). Fifteen articles had to be excluded because they did not fulfill the other aforementioned inclusion criteria. In total, we included 82 articles published between 1938 and 2015 and reporting on a total of 157 patients. Apart from one pilot study, all articles were case reports or small retrospective case series. The pilot study of the Children’s Cancer Group (CCG), CCG-945, enrolled 18 patients with newly diagnosed primary spinal high-grade astrocytomas, including four children with glioblastoma multiforme [4]. All case series focused on malignant spinal cord tumors with a maximum of 14 glioblastoma cases. The procedure of publication retrieval and in- and exclusion of cases is displayed in a PRISMA (Preferred Reporting Items for Systematic Reviews and Meta-Analyses) flow chart (Fig 1) [5].

#### Data extraction

From the eligible articles, the following variables were recorded on a standard data extraction form:

(1) Overall survival
(2) Pattern of failure (local versus distant)
(3) year of publication
(4) age at initial diagnosis
(5) gender
(6) histology
(7) site of primary tumor, compiled into
  - cervical spinal cord
  - cervicothoracic spinal cord
  - thoracic spinal cord
  - thoracolumbar spinal cord
  - lumbar spinal cord
  - cervicolumbar spinal cord / holocord
(9) treatment after diagnosis, categorized as
  - not reported or best supportive care
  - surgery only
  - chemotherapy only
  - radiotherapy only
  - radiotherapy + chemotherapy
  - surgery + radiotherapy
  - surgery + chemotherapy
  - surgery + chemotherapy + radiotherapy

### Statistics

The follow-up time of the patients was quantified according to the method suggested by Schemper and Smith [6]. Survival times were calculated from the time of diagnosis of primary spinal GB / GS. Death resulting from any cause was defined as event for overall survival (OS). The Kaplan-Meier method was used to estimate OS. Figures were rounded to the nearest integer whenever appropriate.

#### Univariate analysis

OS was estimated using the Kaplan-Meier method. Survival plots relating to categorical variables were compared by means of the log rank test. A difference was assumed if the p-value was less than 0.05.

#### Multivariable analysis

In addition, a multivariable cox proportional hazards analysis including the four categorical variables age at diagnosis (cutoff 60 years), time period of publication (cutoff 1980), tumor location (involvement of the cervical spinal cord, yes or no) and extent of resection (gross total resection (GTR), yes or no) was performed using a forward stepwise selection; predictors were removed when their p-value in the likelihood ratio-test was greater than 0.10. Proportional hazards assumptions were checked graphically. For the final models, the estimated hazard ratios of the selected explanatory variables with respective 95% confidence intervals (CI) and p-values of the likelihood ratio test are given. A value of p < 0.05 was considered statistically significant. All analyses were conducted using SPSS, version 20.0 (SPSS Inc., Chicago, IL, USA).

## Results

### Clinical Characteristics

**Fig 2:**
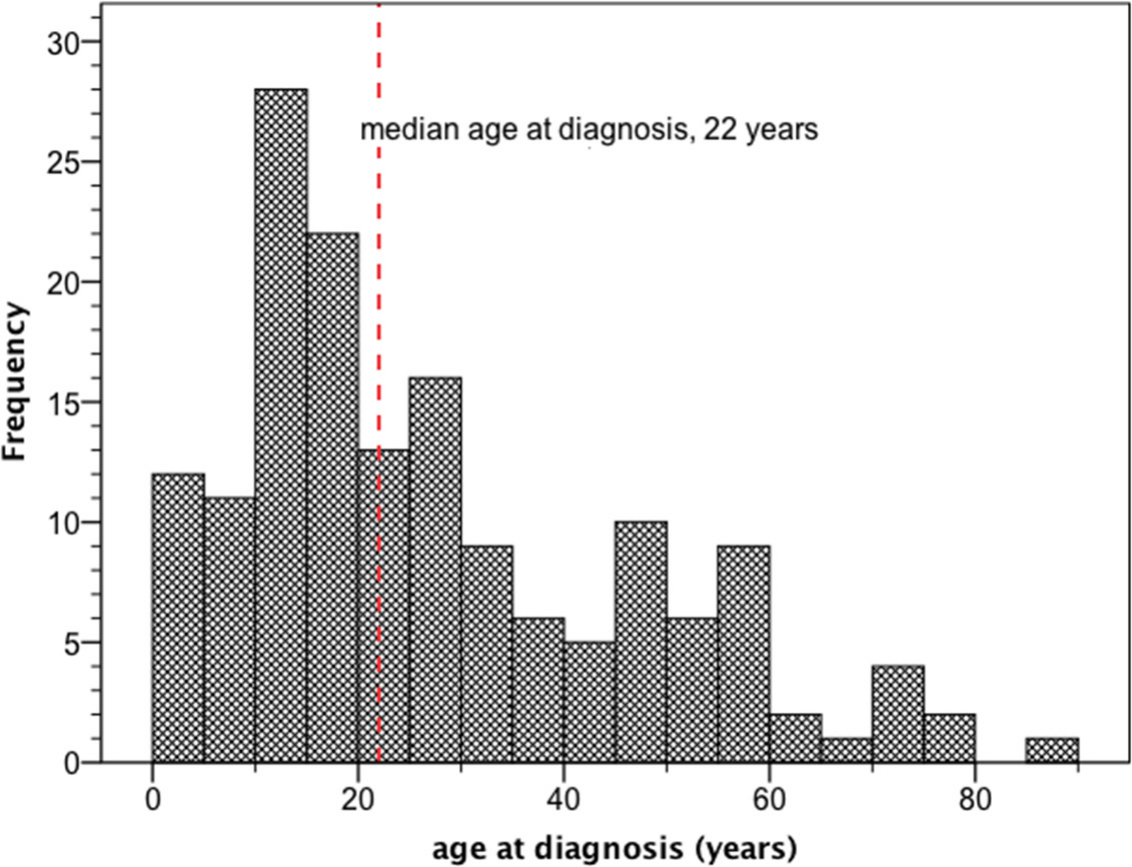
Age distribution of 157 patients with primary spinal origin reported in literature until March 2015. It is displayed in the form of a histogram. Median age at diagnosis was 22 years.

Gender was unknown in 2/157 cases (1%). 84/155 patients (54%) were male and 71/155 (46%) female. Median follow-up time was 96 ± 43 months. The 95% confidence interval of the median follow-up time reached from 13 to 179 months. Age at initial diagnosis was reported for all patients. Median age at initial diagnosis was 22 years (range 0–88 years) (Fig 2). Histopathological diagnosis revealed GB in 155/157 cases (99%). Two patients (1%) had GS. Sixty-six patients (42%) were children or adolescents (< 18 years). Only 10 patients (6%) were 60 years old or older. The tumors affected the cervical spinal cord in 75/157 cases (48%). In 14/157 cases (9%) the patients received best supportive care only. In the remaining 143/157 cases (91%) more intensive treatments were applied. Nineteen patients (13%) underwent surgery only, fourteen patients (10%) received exclusively radiotherapy and one patient (1%) was treated with no more than chemotherapy. 109 (76%) patients underwent a combination of different treatments (surgery + chemotherapy, n = 4; surgery + radiotherapy, n = 47; chemotherapy + radiotherapy, n = 15; surgery + chemotherapy + radiotherapy, n = 43). In 5/157 cases (3%) it remained unclear whether initial GTR was achieved or not. 36 of the remaining 152 patients (24%) underwent initial GTR, whereas 116 (76%) did not. The proportion of patients in whom GTR was initially achieved remained relatively constant over decades (before 1980, 4/21 patients (19%); between 1980 and 2015, 32/131 patients (24%)). In 8/157 cases (5%) it remained unclear whether adjuvant radiotherapy was given or not. 119 of the remaining 149 patients (80%) underwent irradiation, whereas 30 (20%) did not. The proportion of irradiated patients continued to increase over the decades (before 1980, 14/23 patients (61%); between 1980 and 2015, 105/126 patients (83%)). In 31/157 cases (20%) it remained unclear whether adjuvant chemotherapy was given or not. Only half of the remaining 126 patients (n = 63 patients, 50%) received chemotherapy. The proportion of patients who received chemotherapy continued to increase over the decades (before 1980, 1/23 patients (4%); between 1980 and 2015, 62/103 patients (60%)). The total radiation doses ranges from 18 Gy to 75 Gy with an average value of 49.2 Gy. In total 63 patients received chemotherapy (Table 1). The most frequent chemotherapeutic agent was temozolomide, it was used in 30/63 cases (48%). The second most commonly used drug was the angiogenesis inhibitor bevacizumab, which was prescribed in 11/63 cases (18%).

**Table 1:** Clinical characteristics and treatments of 157 patients with primary spinal GB / GS reported in literature until March 2015.

	total	%
Gender not specified	2/157	1
Male	84/155	54
Female	71/155	46
Children (< 18 years)	57/157	36
Adults (> = 18 years)	100/157	64
GBM	155/157	99
GS	2/157	1
Surgery only	19/143	13
Radiotherapy only	14/143	10
Chemotherapy only	1/143	1
Combination of different treatments	109/143	76
Surgery + chemotherapy	4/109	4
Surgery + radiotherapy	47/109	43
Chemotherapy + radiotherapy	15/109	14
Surgery + chemotherapy + radiotherapy	43/109	39
Supportive care only	14/157	9
Death	130/157	83

GB: Glioblastoma, GS: Gliosarcoma

#### Overall survival from initial diagnosis

**Fig 3:**
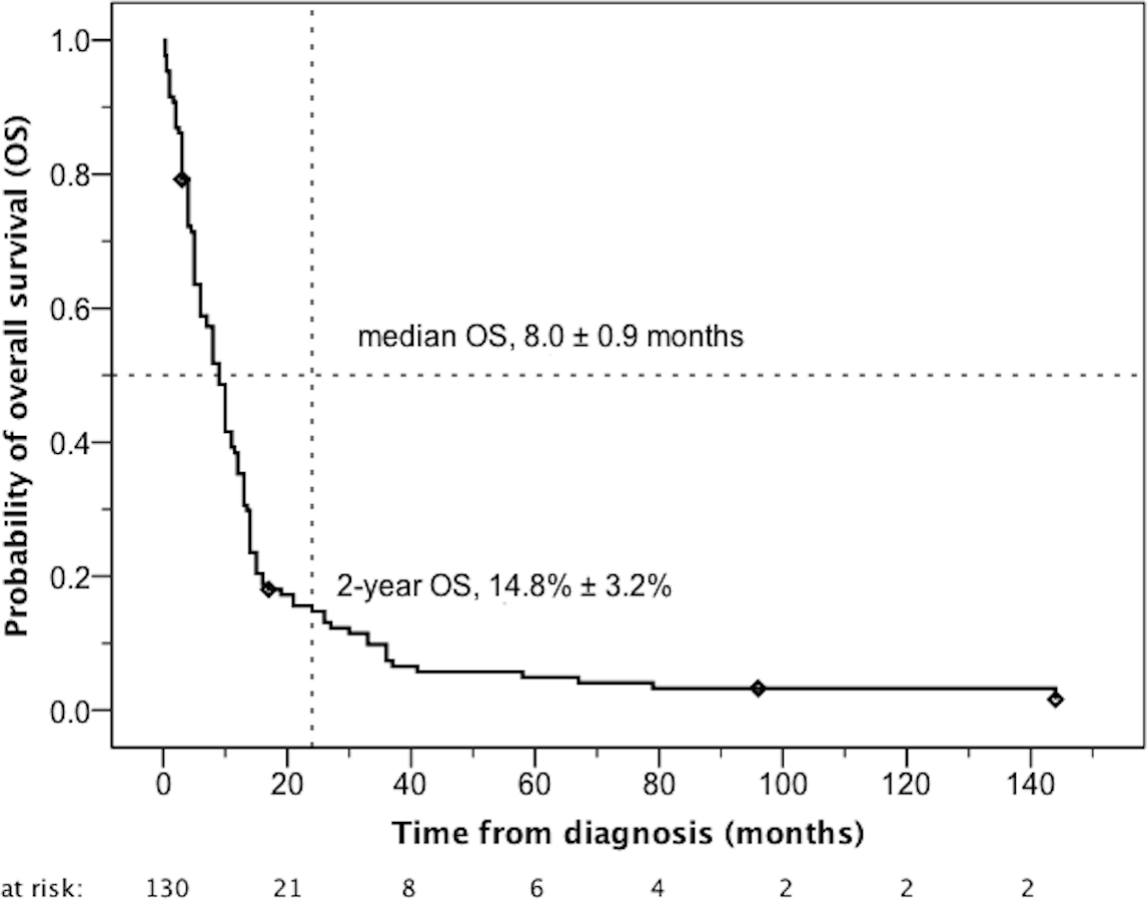
Overall survival of 130 patients with primary spinal glioblastomas /- sarcomas reported in literature until March 2015. In 27 of the 157 patients (17%) survival data were not available.

In 130/157 cases sufficient information was available to estimate overall survival from initial diagnosis (OS). Median OS of these 130 patients was 9.0 ± 0.8 months. Only 15% ± 3% of the patients were still alive two years after the initial diagnosis (Fig 3).

### Potential prognostic factors for overall survival

#### Univariate analysis using the Kaplan Meier method and log rank test

**Fig 4:**
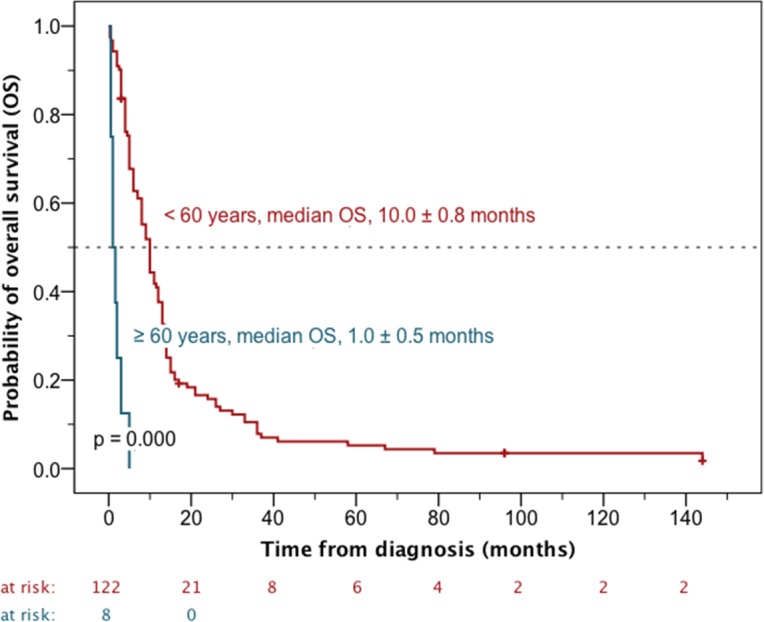
Impact of age on overall survival (OS). On univariate analysis the older patients (≥ 60 years at initial diagnosis) had significantly shorter survival times (p < 0.0001).

**Fig 5:**
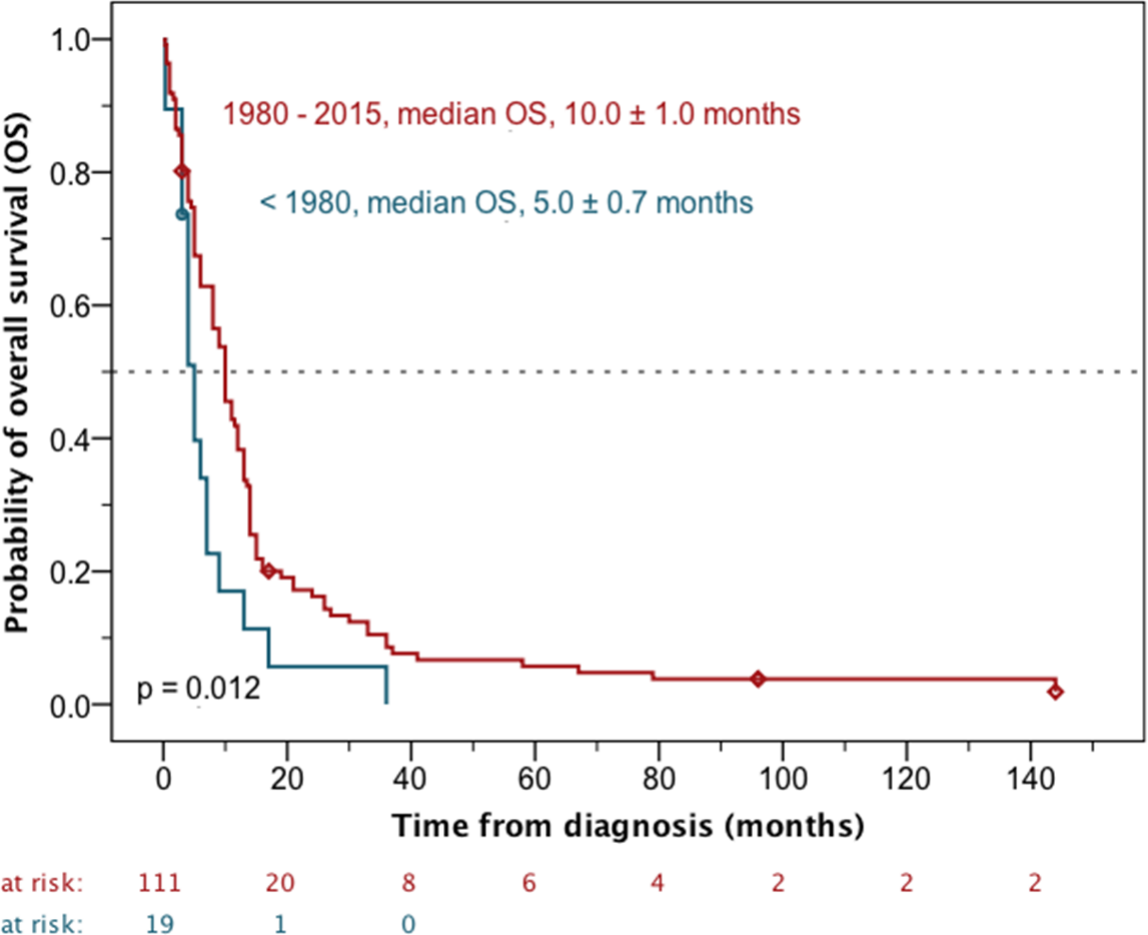
Overall survival (OS) according to the publications before and after 1980. A modest increase in treatment success has been achieved over recent decades.

On univariate analysis age at initial diagnosis and the time period when the medical treatment was performed influenced OS. Advanced age was related to a worse prognosis. Patients younger than 60 years of age had a median overall survival of 10.0 ± 0.8 months, whereas it was only 1.0 ± 0.5 months in the 60-plus cohort (p < 0.0001) (Fig 4). A similar effect was observed when using 50 years as cut-off (p = 0.043). In contrast, children (< 18 years) and adults (≥ 18 years) showed comparable outcomes (p = 0.398). Patients treated before 1980 had shorter survival times (median OS, 5.0 ± 0.7 months) than patients treated between 1980 and 2015 (median OS, 10.0 ± 1.0 months, p = 0.012) (Fig 5). In contrast, OS was not influenced by initial gross total resection (GTR) (p = 0.094), gender (p = 0.311) or cervical spinal cord affection (p = 0.057) (Table 2).

**Table 2:** Evaluation of potential risk factors for overall survival using Kaplan Meier method and log rank test.

Subgroup	Number of patients	Deaths	Median OS (months)	SE	Log rank p =
male	70	65	8.0	1.5	0.311
female	58	57	10.0	0.9	0.311
< 18 years at ID	57	54	9.0	1.5	0.398
> = 18 years at ID	73	70	9.0	1.3	0.398
< 50 years at ID	108	102	10.0	1.0	0.043
> = 50 years at ID	22	22	3.0	0.7	0.043
< 60 years at ID	122	116	10.0	0.8	< 0.0001
> = 60 years at ID	8	8	1.0	0.5	< 0.0001
Initial GTR	34	30	12.0	2.5	0.094
no GTR	93	91	8.0	1.1	0.094
cervical affection	65	63	8.0	1.4	0.057
no cervical affection	65	61	10.0	1.2	0.057
time period of treatment					
before 1980	19	18	5.0	0.7	0.012
1980–2015	111	106	10.0	1.0	0.012

OS: overall survival, SE: standard error, ID: initial diagnosis, GTR: gross total resection

#### Multivariable analysis using cox regression

Multivariable analysis only identified age at diagnosis < 60 years (hazard ratio = 0.097, 95% CI: 0.043–0.217, p < 0.0001) and the time period of publication (< 1980, hazard ratio 1.996, 95% CI: 1.182–3.372, p = 0.010) as independent predictors of survival. In contrast, the extent of resection (GTR) and tumor location (cervical affection) did not reach statistical significance.

## Discussion

### General aspects

In adults, glioblastoma is the most frequent malignant brain tumor. Metastases to the spinal cord or primary spinal location are rare. In two previous meta-analyses we focused on patients with metastatic disease [7, 8]. Now, we generated a large database of glioblastoma cases with primary spinal location. 157 patients reported in the literature until March 2015 met our inclusion criteria and were evaluated in detail. We focused our analysis on patient characteristics, treatments, prognosis and factors associated with survival.

A similar population-based analysis using data from the population-based cancer registries of the Surveillance, Epidemiology and End Results (SEER) program from 1973–2007 had already been published in 2012. However, the authors, Adams et al., did not exclusively focus on glioblastomas (WHO grade IV) but also included spinal anaplastic astrocytomas (WHO grade III) into their study. Moreover, the number of glioblastoma cases was much smaller (n = 59) [9].

### Which age group is at risk for primary spinal glioblastoma?

Interestingly, our data suggest that primary spinal glioblastomas / -sarcomas mainly affect younger patients. Median age at diagnosis in our cohort was 22 years. Only 10/157 patients (6%) were 60 years old or older. This result matches well with the findings of Adams et al. who calculated a mean age of 35 years in their cohort [9]. In contrast, the median age of patients with primary intracranial glioblastomas is much higher. For example in our previous meta-analyses, the median age at initial diagnosis was 42 years [7] and 44 years [8]. Furthermore, in the well known European cohort evaluated by Stupp et al. in order to demonstrate the benefit of temozolomide, the median age at diagnosis was 56 years [10]. Older data from the Los Angeles County Cancer Surveillance Program showed an even higher median age at diagnosis of intracranial glioblastoma at approximately 65 years [11].

### What is the prognosis of patients with primary spinal glioblastomas?

In the 1980s high-grade gliomas of spinal origin were thought to have the same outcome as their intracranial counterparts [12]. However, the prognosis of the latter has significantly improved over the past decades and the question arises whether this statement is still true. Depending on tumor biology, particularly on the methylation status of the O6-methylguanine-DNA methyltransferase (MGMT) promotor, intracranial glioblastomas are now characterized by median survival rates ranging between 15 and 22 months when patients are treated with an up-to-date multidisciplinary approach. This consists of surgical resection and radiotherapy (60 Gy) combined with concomitant and adjuvant treatment with temozolomide [13]. According to this, in a recent article, the estimated „average survival”of patients with spinal glioblastomas was 18 months [14]. However, an explanation of how this figure was calculated was not provided.

In contrast, median survival in our cohort was clearly worse (9.0 ± 0.8 months). Only 15 ± 3% of patients were still alive two years after the initial diagnosis. In the analyses of Adams et al. [9] and Santi et al. [15] the median survival of patients with spinal glioblastomas was 10 months which is in line with our findings. Even when focusing on the cases which were reported after 2005, i.e. on the patients, who should have been treated in accordance with modern treatment principles, median survival in our cohort is still remarkably poor (11.0 ± 1.1 months).

### Which factors impact on survival?

In the analysis of Adams et al. only the histological subtype, age at diagnosis (particularly when categorizing the patients into children and adults), sex, and the extent of resection were associated with survival in a final multivariable model. Of note, this cohort mainly contained patients with primary spinal anaplastic astrocytomas, whereas the number of GB patients was limited (n = 59) [9]. Focusing on WHO grade IV tumors and based on a considerably larger number of cases (n = 157) the present analysis paints a partially different picture.

In contrast to the findings of Adams et al. [9] female gender was not related to poorer survival. Our result is supported by a landmark study on prognostic factors in malignant glioma patients with an intracranial tumor site. The authors, Curran et al., assessed a total of 1578 patients entered in three consecutive Radiation Therapy Oncology Group malignant glioma trials and developed a prognostic model in which gender did not have any relevance [16].

The question whether radical resection is beneficial for the outcome of patients with spinal malignant gliomas is controversial. In the present study the patients with initial gross total resection (GTR) did not show longer survival. In contrast, the data from the SEER database showed a significant association of the extent of surgical resection with survival in both uni- and multivariable analyses [9]. However, a previous smaller series failed to demonstrate an impact of radical resection on survival [17]. In an Italian study surgical treatment did not ameliorate the neurological status; instead, in the majority of cases, it prompted a worsening of the deficits [18]. In the setting of a pre-existing tumor-related complete neurological deficit cordectomy may be a valuable therapeutic alternative. Nevertheless, this radical approach is only feasible if the upper thoracic and cervical spinal cord are intact. Some authors suppose that cordectomy may provide longer survival than would otherwise be achievable with more radiation or the addition of chemotherapy [14]. In our series we identified 6 patients undergoing cordectomy. Indeed, all but one [19] showed excellent survival rates ranging between 14 and 144 months [20–22, 14].

In our study children and adults with primary spinal GB / GS shared the same outcomes whereas the analysis of Adams et al. demonstrated a difference between the pediatric and adult age groups and their survival rates. Here, the median survival of adult patients in comparison to the pediatric cohort was significantly better at 16 versus 9 months [9]. Due to these contradicting study results the question whether pediatric and adult tumors are characterized by different prognoses remains unanswered. In two previous studies of our working group focusing on metastatic GB / GS we encountered the same problem [7, 8].

Of note, in the present analysis age (as categorical variable) became a powerful prognostic factor in both uni- and multivariable analysis when assessing a different cut-off. On univariate analysis the median overall survival of patients younger than 60 years of age was ten times longer than that of the 60-plus cohort. A similar effect was observed with a cut-off of 50 years. These findings are very plausible as in the setting of primary intracranial disease old age was already identified as one of the pretreatment variables most predictive of survival outcome some decades ago [16].

In the present analysis, the second independent prognostic factor for survival was the time period in which the patients underwent treatment. In univariate analysis, the patients treated before 1980 had shorter survival times than the patients treated between 1980 and 2015, respectively. A possible explanation for this may be that the use of adjuvant treatment (radio- and chemotherapy) significantly increased from the time before 1980 until present. Of note, this explanation is highly speculative as prospective studies demonstrating the benefit of adjuvant radiotherapy in spinal cord malignant gliomas are absent [23, 24, 9]. Based on an older retrospective analysis Shirato et al. recommended conventional radiotherapy after less than total resection of spinal cord astrocytomas [25]. However, their conclusions have to be treated with caution since they treated very low patient numbers.

To the best of our knowledge the same applies to the use of adjuvant temozolomide. Interestingly, immunotherapy with the angiogenesis inhibitor bevacizumab was also frequently administered. However, two large prospective randomized trials recently revealed that bevacizumab has no impact on the survival of GB patients [26, 27].

### Limitations inherent to systematic reviews

First of all, there is certainly a selection bias, because the cases reported in the literature might have been published due to their rare or uncommon occurrences and outcomes. Furthermore, not all data were available for each individual patient, so that the time course of these diseases could not always be reconstructed accurately. This is reflected by different patient numbers in patient characteristics and survival analyses.

#### Statistical limitations

In this study, the potential benefit of radio- and/or chemotherapy or other treatment-related factors for survival was not investigated. The reason for this is that the patients in our cohort received individualized treatments to the effect that these factors were unknown at the beginning of survival time. To investigate a variable that is still elusive at the beginning of survival time or that changes over time, time-dependent Cox regression could be used. Therefore this procedure requires particularly detailed information about the starting date of therapy [28], which was generally not provided by these case reports in the literature. Alternatively, the effect of a time-varying treatment could be investigated using marginal structural models or structural nested failure time models.

## Conclusions

These rare tumors of the spinal cord are associated with a dismal prognosis whereby only modest treatment progress has been achieved over the last decades. The characteristics and treatments of primary spinal glioblastomas should be entered into a central registry in order to gain more information about the ideal treatment approach in the future.

## Supporting Information

S1 PRISMA Checklist This analysis was performed according to the PRISMA (Preferred Reporting Items for Systematic reviews and Meta-Analysis) guidelines. (DOCX)

S1 Search Methods Search Strategy and Terms used to identify eligible articles. (TIFF)

S1 Dataset Available data on clinical characteristics, treatment and outcome for each individual patient. No.: number, OS ID: overall survival from initial diagnosis.
(XLSX)

S1 References List of references of the applied Data including DOI or PMID for each article if available. (DOCX)
