# Supplementary figures and images for: A Systematic Review on the Characteristics, Treatments and Outcomes of the Patients with Primary Spinal Glioblastomas or Gliosarcomas Reported in Literature until March 2015
Source: PLoS One. 2016 Feb 9;11(2):e0148312. doi: 10.1371/journal.pone.0148312 (PMC4747550; doi:10.1371/journal.pone.0148312)

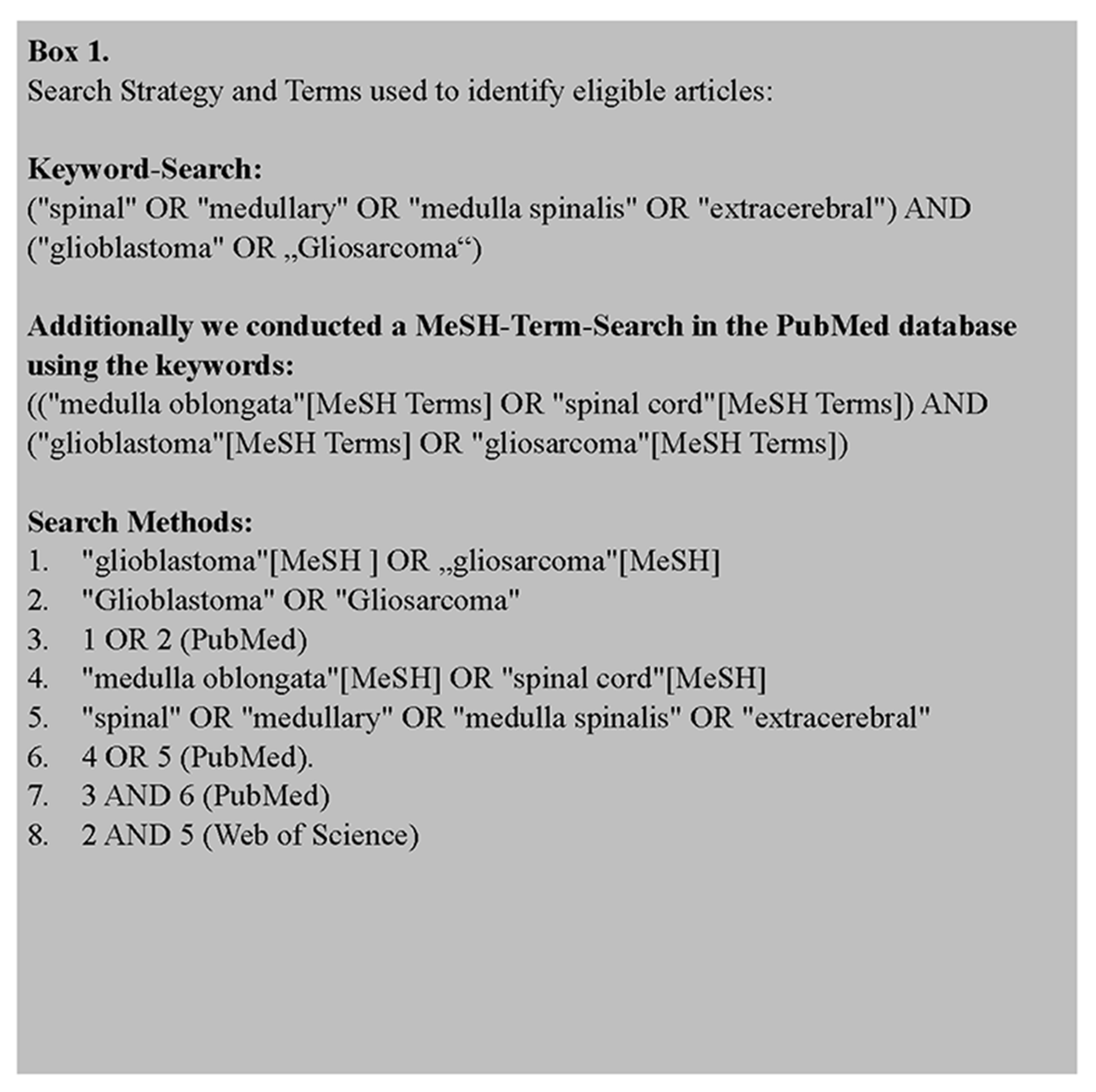

Supplement: S1 Search Methods — (TIFF) [file pone.0148312.s002.tiff]
