# Supplementary material for: A Systematic Review on the Characteristics, Treatments and Outcomes of the Patients with Primary Spinal Glioblastomas or Gliosarcomas Reported in Literature until March 2015
Source: PLoS One. 2016 Feb 9;11(2):e0148312. doi: 10.1371/journal.pone.0148312 (PMC4747550; doi:10.1371/journal.pone.0148312)
Supplement: S1 References — (DOCX) [file pone.0148312.s004.docx]

1. [Ahn SJ](http://www.ncbi.nlm.nih.gov/pubmed/?term=Ahn%20SJ%5BAuthor%5D&cauthor=true&cauthor_uid=22977336), [Kim IO](http://www.ncbi.nlm.nih.gov/pubmed/?term=Kim%20IO%5BAuthor%5D&cauthor=true&cauthor_uid=22977336). Spinal cord glioblastoma induced by radiation therapy of nasopharyngeal rhabdomyosarcoma with MRI findings: case report. Korean J Radiol. 2012 Sep-Oct;13(5):652-7. PMID: 22977336
2. [Allen JC](http://www.ncbi.nlm.nih.gov/pubmed/?term=Allen%20JC%5BAuthor%5D&cauthor=true&cauthor_uid=9452226), [Aviner S](http://www.ncbi.nlm.nih.gov/pubmed/?term=Aviner%20S%5BAuthor%5D&cauthor=true&cauthor_uid=9452226), [Yates AJ](http://www.ncbi.nlm.nih.gov/pubmed/?term=Yates%20AJ%5BAuthor%5D&cauthor=true&cauthor_uid=9452226), [Boyett JM](http://www.ncbi.nlm.nih.gov/pubmed/?term=Boyett%20JM%5BAuthor%5D&cauthor=true&cauthor_uid=9452226), [Cherlow JM](http://www.ncbi.nlm.nih.gov/pubmed/?term=Cherlow%20JM%5BAuthor%5D&cauthor=true&cauthor_uid=9452226), [Turski PA](http://www.ncbi.nlm.nih.gov/pubmed/?term=Turski%20PA%5BAuthor%5D&cauthor=true&cauthor_uid=9452226), [Epstein F](http://www.ncbi.nlm.nih.gov/pubmed/?term=Epstein%20F%5BAuthor%5D&cauthor=true&cauthor_uid=9452226), [Finlay JL](http://www.ncbi.nlm.nih.gov/pubmed/?term=Finlay%20JL%5BAuthor%5D&cauthor=true&cauthor_uid=9452226). Treatment of high-grade spinal cord astrocytoma of childhood with "8-in-1" chemotherapy and radiotherapy: a pilot study of CCG-945. Children's Cancer Group. J Neurosurg. 1998 Feb;88(2):215-20. PMID: 9452226
3. Ando K, Matsuyama Y, Sakai Y, Imagama S, Ito Z, Wakao N, Ishiguro N. Cervical intramedullary glioblastoma with intracranial dissemination: description of a rapidly progressing case and a literature review. *J. Musculoskelet. Res.* 13, 43 (2010). DOI: 10.1142/S0218957710002442
4. [Andrews AA](http://www.ncbi.nlm.nih.gov/pubmed/?term=Andrews%20AA%5BAuthor%5D&cauthor=true&cauthor_uid=205201), [Enriques L](http://www.ncbi.nlm.nih.gov/pubmed/?term=Enriques%20L%5BAuthor%5D&cauthor=true&cauthor_uid=205201), [Renaudin J](http://www.ncbi.nlm.nih.gov/pubmed/?term=Renaudin%20J%5BAuthor%5D&cauthor=true&cauthor_uid=205201), [Tomiyasu U](http://www.ncbi.nlm.nih.gov/pubmed/?term=Tomiyasu%20U%5BAuthor%5D&cauthor=true&cauthor_uid=205201). Spinal intramedullary glioblastoma with intracranial seeding. Report of a case. Arch Neurol. 1978 Apr;35(4):244-5. PMID: 205201
5. [Arumugasamy N](http://www.ncbi.nlm.nih.gov/pubmed/?term=Arumugasamy%20N%5BAuthor%5D&cauthor=true&cauthor_uid=4351105), [Tarkington JA](http://www.ncbi.nlm.nih.gov/pubmed/?term=Tarkington%20JA%5BAuthor%5D&cauthor=true&cauthor_uid=4351105). Primary glioblastoma multiforme of the spinal cord in infancy and childhood. Med J Malaya. 1972 Dec;27(2):136-41.PMID: 4351105
6. [Asano N](http://www.ncbi.nlm.nih.gov/pubmed/?term=Asano%20N%5BAuthor%5D&cauthor=true&cauthor_uid=1701860), [Kitamura K](http://www.ncbi.nlm.nih.gov/pubmed/?term=Kitamura%20K%5BAuthor%5D&cauthor=true&cauthor_uid=1701860), [Seo Y](http://www.ncbi.nlm.nih.gov/pubmed/?term=Seo%20Y%5BAuthor%5D&cauthor=true&cauthor_uid=1701860), [Mukai K](http://www.ncbi.nlm.nih.gov/pubmed/?term=Mukai%20K%5BAuthor%5D&cauthor=true&cauthor_uid=1701860), [Soga T](http://www.ncbi.nlm.nih.gov/pubmed/?term=Soga%20T%5BAuthor%5D&cauthor=true&cauthor_uid=1701860), [Hondo H](http://www.ncbi.nlm.nih.gov/pubmed/?term=Hondo%20H%5BAuthor%5D&cauthor=true&cauthor_uid=1701860), [Matsumoto K](http://www.ncbi.nlm.nih.gov/pubmed/?term=Matsumoto%20K%5BAuthor%5D&cauthor=true&cauthor_uid=1701860). Spinal cord glioblastoma multiforme with intracranial dissemination-case report. Neurol Med Chir (Tokyo). 1990 Jul;30(7):489-94. PMID: 1701860
7. [Banczerowski P](http://www.ncbi.nlm.nih.gov/pubmed/?term=Banczerowski%20P%5BAuthor%5D&cauthor=true&cauthor_uid=12690787), [Simó M](http://www.ncbi.nlm.nih.gov/pubmed/?term=Sim%C3%B3%20M%5BAuthor%5D&cauthor=true&cauthor_uid=12690787), [Sipos L](http://www.ncbi.nlm.nih.gov/pubmed/?term=Sipos%20L%5BAuthor%5D&cauthor=true&cauthor_uid=12690787), [Slowik F](http://www.ncbi.nlm.nih.gov/pubmed/?term=Slowik%20F%5BAuthor%5D&cauthor=true&cauthor_uid=12690787), [Benoist G](http://www.ncbi.nlm.nih.gov/pubmed/?term=Benoist%20G%5BAuthor%5D&cauthor=true&cauthor_uid=12690787), [Veres R](http://www.ncbi.nlm.nih.gov/pubmed/?term=Veres%20R%5BAuthor%5D&cauthor=true&cauthor_uid=12690787). Primary intramedullary glioblastoma multiforme of the spinal cord: report of eight cases. Ideggyogy Sz. 2003 Jan 20;56(1-2):28-32. PMID: 12690787
8. [Barbagallo GM](http://www.ncbi.nlm.nih.gov/pubmed/?term=Barbagallo%20GM%5BAuthor%5D&cauthor=true&cauthor_uid=12450287), [Lanzafame S](http://www.ncbi.nlm.nih.gov/pubmed/?term=Lanzafame%20S%5BAuthor%5D&cauthor=true&cauthor_uid=12450287), [Nicol eG](http://www.ncbi.nlm.nih.gov/pubmed/?term=Nicol%20eG%5BAuthor%5D&cauthor=true&cauthor_uid=12450287), [Platania N](http://www.ncbi.nlm.nih.gov/pubmed/?term=Platania%20N%5BAuthor%5D&cauthor=true&cauthor_uid=12450287), [Albanese V](http://www.ncbi.nlm.nih.gov/pubmed/?term=Albanese%20V%5BAuthor%5D&cauthor=true&cauthor_uid=12450287). Primary C1-2, intradural, extramedullary meningeal sarcoma with glial fibrillary acidic protein-immunoreactive components: a spinal gliosarcoma? Case report and review of the literature. J Neurosurg. 2002 Mar;96(2 Suppl):230-5. PMID: 12450287
9. [Battaglia S](http://www.ncbi.nlm.nih.gov/pubmed/?term=Battaglia%20S%5BAuthor%5D&cauthor=true&cauthor_uid=24299936), [Albini Riccioli L](http://www.ncbi.nlm.nih.gov/pubmed/?term=Albini%20Riccioli%20L%5BAuthor%5D&cauthor=true&cauthor_uid=24299936), [Bartiromo F](http://www.ncbi.nlm.nih.gov/pubmed/?term=Bartiromo%20F%5BAuthor%5D&cauthor=true&cauthor_uid=24299936), [Galassi E](http://www.ncbi.nlm.nih.gov/pubmed/?term=Galassi%20E%5BAuthor%5D&cauthor=true&cauthor_uid=24299936), [Marliani AF](http://www.ncbi.nlm.nih.gov/pubmed/?term=Marliani%20AF%5BAuthor%5D&cauthor=true&cauthor_uid=24299936), [Leonardi M](http://www.ncbi.nlm.nih.gov/pubmed/?term=Leonardi%20M%5BAuthor%5D&cauthor=true&cauthor_uid=24299936). Childhood spinal glioblastoma multiforme with intracranial dissemination. A case report. Neuroradiol J. 2007 Oct 31;20(5):500-3. Epub 2007 Oct 31. PMID: 24299936
10. [Bonde V](http://www.ncbi.nlm.nih.gov/pubmed/?term=Bonde%20V%5BAuthor%5D&cauthor=true&cauthor_uid=18329879), [Balasubramaniam S](http://www.ncbi.nlm.nih.gov/pubmed/?term=Balasubramaniam%20S%5BAuthor%5D&cauthor=true&cauthor_uid=18329879), [Goel A](http://www.ncbi.nlm.nih.gov/pubmed/?term=Goel%20A%5BAuthor%5D&cauthor=true&cauthor_uid=18329879). Glioblastoma multiforme of the conus medullaris with holocordal spread. J Clin Neurosci. 2008 May;15(5):601-3. PMID: 18329879
11. [Caroli E](http://www.ncbi.nlm.nih.gov/pubmed/?term=Caroli%20E%5BAuthor%5D&cauthor=true&cauthor_uid=15838531), [Salvati M](http://www.ncbi.nlm.nih.gov/pubmed/?term=Salvati%20M%5BAuthor%5D&cauthor=true&cauthor_uid=15838531), [Ferrante L](http://www.ncbi.nlm.nih.gov/pubmed/?term=Ferrante%20L%5BAuthor%5D&cauthor=true&cauthor_uid=15838531). Spinal glioblastoma with brain relapse in a child: clinical considerations. Spinal Cord. 2005 Sep;43(9):565-7. PMID: 15838531
12. [Carstens PH](http://www.ncbi.nlm.nih.gov/pubmed/?term=Carstens%20PH%5BAuthor%5D&cauthor=true&cauthor_uid=7605106), [Johnson GS](http://www.ncbi.nlm.nih.gov/pubmed/?term=Johnson%20GS%5BAuthor%5D&cauthor=true&cauthor_uid=7605106), [Jelsma LF](http://www.ncbi.nlm.nih.gov/pubmed/?term=Jelsma%20LF%5BAuthor%5D&cauthor=true&cauthor_uid=7605106).Spinal gliosarcoma: a light, immunohistochemical and ultrastructural study.Ann Clin Lab Sci. 1995 May-Jun;25(3):241-6. PMID: 7605106
13. [de Castro-Costa CM](http://www.ncbi.nlm.nih.gov/pubmed/?term=de%20Castro-Costa%20CM%5BAuthor%5D&cauthor=true&cauthor_uid=8002810), [de Araújo RW](http://www.ncbi.nlm.nih.gov/pubmed/?term=de%20Ara%C3%BAjo%20RW%5BAuthor%5D&cauthor=true&cauthor_uid=8002810), [de Arruda MA](http://www.ncbi.nlm.nih.gov/pubmed/?term=de%20Arruda%20MA%5BAuthor%5D&cauthor=true&cauthor_uid=8002810), [de Araújo PM](http://www.ncbi.nlm.nih.gov/pubmed/?term=de%20Ara%C3%BAjo%20PM%5BAuthor%5D&cauthor=true&cauthor_uid=8002810), [de Figueiredo EG](http://www.ncbi.nlm.nih.gov/pubmed/?term=de%20Figueiredo%20EG%5BAuthor%5D&cauthor=true&cauthor_uid=8002810). Increased intracranial pressure in a case of spinal cervical glioblastoma multiforme. Analysis of these two rare conditions. Arq Neuropsiquiatr. 1994 Mar;52(1):64-8. PMID: 8002810
14. [Chamberlain MC](http://www.ncbi.nlm.nih.gov/pubmed/?term=Chamberlain%20MC%5BAuthor%5D&cauthor=true&cauthor_uid=20680397), [Johnston SK](http://www.ncbi.nlm.nih.gov/pubmed/?term=Johnston%20SK%5BAuthor%5D&cauthor=true&cauthor_uid=20680397). Recurrent spinal cord glioblastoma: salvage therapy with bevacizumab. J Neurooncol. 2011 May;102(3):427-32. PMID: 20680397
15. [Choi WC](http://www.ncbi.nlm.nih.gov/pubmed/?term=Choi%20WC%5BAuthor%5D&cauthor=true&cauthor_uid=19159817), [Lee JH](http://www.ncbi.nlm.nih.gov/pubmed/?term=Lee%20JH%5BAuthor%5D&cauthor=true&cauthor_uid=19159817), [Lee SH](http://www.ncbi.nlm.nih.gov/pubmed/?term=Lee%20SH%5BAuthor%5D&cauthor=true&cauthor_uid=19159817). Spinal cord glioblastoma multiforme of conus medullaris masquerading as high lumbar disk herniation. Surg Neurol. 2009 Feb;71(2):234-7; discussion 237. PMID: 19159817
16. [Ciappetta P](http://www.ncbi.nlm.nih.gov/pubmed/?term=Ciappetta%20P%5BAuthor%5D&cauthor=true&cauthor_uid=1847741), [Salvati M](http://www.ncbi.nlm.nih.gov/pubmed/?term=Salvati%20M%5BAuthor%5D&cauthor=true&cauthor_uid=1847741), [Capoccia G](http://www.ncbi.nlm.nih.gov/pubmed/?term=Capoccia%20G%5BAuthor%5D&cauthor=true&cauthor_uid=1847741), [Artico M](http://www.ncbi.nlm.nih.gov/pubmed/?term=Artico%20M%5BAuthor%5D&cauthor=true&cauthor_uid=1847741), [Raco A](http://www.ncbi.nlm.nih.gov/pubmed/?term=Raco%20A%5BAuthor%5D&cauthor=true&cauthor_uid=1847741), [Fortuna A](http://www.ncbi.nlm.nih.gov/pubmed/?term=Fortuna%20A%5BAuthor%5D&cauthor=true&cauthor_uid=1847741). Spinal glioblastomas: report of seven cases and review of the literature. Neurosurgery. 1991 Feb;28(2):302-6. PMID: 1847741
17. [Cohen AR](http://www.ncbi.nlm.nih.gov/pubmed/?term=Cohen%20AR%5BAuthor%5D&cauthor=true&cauthor_uid=2909688), [Wisoff JH](http://www.ncbi.nlm.nih.gov/pubmed/?term=Wisoff%20JH%5BAuthor%5D&cauthor=true&cauthor_uid=2909688), [Allen JC](http://www.ncbi.nlm.nih.gov/pubmed/?term=Allen%20JC%5BAuthor%5D&cauthor=true&cauthor_uid=2909688), [Epstein F](http://www.ncbi.nlm.nih.gov/pubmed/?term=Epstein%20F%5BAuthor%5D&cauthor=true&cauthor_uid=2909688). Malignant astrocytomas of the spinal cord. J Neurosurg. 1989 Jan;70(1):50-4. PMID: 2909688
18. [Eade OE](http://www.ncbi.nlm.nih.gov/pubmed/?term=Eade%20OE%5BAuthor%5D&cauthor=true&cauthor_uid=5563877), [Urich H](http://www.ncbi.nlm.nih.gov/pubmed/?term=Urich%20H%5BAuthor%5D&cauthor=true&cauthor_uid=5563877). Metastasising gliomas in young subjects. J Pathol. 1971 Apr;103(4):245-56. PMID: 5563877
19. KC Eden KC, Dissemination of a glioma of the spinal cord into the leptomeninges. Brain 61,298–310 (1938) DOI: <http://dx.doi.org/10.1093/brain/61.3.298>
20. Elsamaloty H, Zenooz N A, Mossa-Basha M. Glioblastoma multiforme (GBM) of the conus medullaris with brain and brain stem metastases. European Journal of Radiology Extra 58 (2006) 59–62.
21. Fortuna A, Giuffré R. Intramedullary glioblastomas. Neurochirurgia (Stuttg). 1971 Jan;14(1):14-23. PMID: 5100022
22. [Gee TS](http://www.ncbi.nlm.nih.gov/pubmed/?term=Gee%20TS%5BAuthor%5D&cauthor=true&cauthor_uid=23082462), [Ghani AR](http://www.ncbi.nlm.nih.gov/pubmed/?term=Ghani%20AR%5BAuthor%5D&cauthor=true&cauthor_uid=23082462), [Idris B](http://www.ncbi.nlm.nih.gov/pubmed/?term=Idris%20B%5BAuthor%5D&cauthor=true&cauthor_uid=23082462), [Awang MS](http://www.ncbi.nlm.nih.gov/pubmed/?term=Awang%20MS%5BAuthor%5D&cauthor=true&cauthor_uid=23082462). Case report: a rare case of pediatric conus medularis glioblastoma multiforme. Med J Malaysia. 2012 Aug;67(4):438-41. PMID: 23082462
23. [Grisold W](http://www.ncbi.nlm.nih.gov/pubmed/?term=Grisold%20W%5BAuthor%5D&cauthor=true&cauthor_uid=7282456), [Pernetzky G](http://www.ncbi.nlm.nih.gov/pubmed/?term=Pernetzky%20G%5BAuthor%5D&cauthor=true&cauthor_uid=7282456), [Jellinger K](http://www.ncbi.nlm.nih.gov/pubmed/?term=Jellinger%20K%5BAuthor%5D&cauthor=true&cauthor_uid=7282456). Giant-cell glioblastoma of the thoracic cord. Acta Neurochir (Wien). 1981;58(1-2):121-6. PMID: 7282456
24. [Hochart, A](http://apps.webofknowledge.com/OneClickSearch.do?product=UA&search_mode=OneClickSearch&excludeEventConfig=ExcludeIfFromFullRecPage&SID=Z1EHGmE2z8Plt6MyTIN&field=AU&value=Hochart,%20A), [Maurage, CA](http://apps.webofknowledge.com/OneClickSearch.do?product=UA&search_mode=OneClickSearch&excludeEventConfig=ExcludeIfFromFullRecPage&SID=Z1EHGmE2z8Plt6MyTIN&field=AU&value=Maurage,%20CA), [Rocourt, N](http://apps.webofknowledge.com/OneClickSearch.do?product=UA&search_mode=OneClickSearch&excludeEventConfig=ExcludeIfFromFullRecPage&SID=Z1EHGmE2z8Plt6MyTIN&field=AU&value=Rocourt,%20N), [Vinchon, M](http://apps.webofknowledge.com/OneClickSearch.do?product=UA&search_mode=OneClickSearch&excludeEventConfig=ExcludeIfFromFullRecPage&SID=Z1EHGmE2z8Plt6MyTIN&field=AU&value=Vinchon,%20M), [Kerdraon, O](http://apps.webofknowledge.com/OneClickSearch.do?product=UA&search_mode=OneClickSearch&excludeEventConfig=ExcludeIfFromFullRecPage&SID=Z1EHGmE2z8Plt6MyTIN&field=AU&value=Kerdraon,%20O), [Escande, F](http://apps.webofknowledge.com/OneClickSearch.do?product=UA&search_mode=OneClickSearch&excludeEventConfig=ExcludeIfFromFullRecPage&SID=Z1EHGmE2z8Plt6MyTIN&field=AU&value=Escande,%20F), [Grill, J](http://apps.webofknowledge.com/OneClickSearch.do?product=UA&search_mode=OneClickSearch&excludeEventConfig=ExcludeIfFromFullRecPage&SID=Z1EHGmE2z8Plt6MyTIN&field=AU&value=Grill,%20J), [Pick, VK](http://apps.webofknowledge.com/OneClickSearch.do?product=UA&search_mode=OneClickSearch&excludeEventConfig=ExcludeIfFromFullRecPage&SID=Z1EHGmE2z8Plt6MyTIN&field=AU&value=Pick,%20VK), SPONTANEOUS MALIGNANT TRANSFORMATION OF A CERVICAL SPINAL CORD PILOCYTIC ASTROCYTOMA INTO GLIOBLASTOMA IN A CHILD. 16th International Symposium on Pediatric Neuro-Oncology (ISPNO), Jun 28-jul 02, 2014. WOS:000337924200163
25. KARACA F, AFŞAR C U, ERKURT E, TUNALI C, ARSLANTAŞ H S, KOCABAŞ C. [Primary Spinal Glioblastoma Multiforme: A Case Report and Review of The Literature](http://www.jns.dergisi.org/text.php3?id=778).Journal of Neurological Sciences (Turkish) 2014, Volume 31, Number 2, Page(s) 366-376. WOS:000337924200163
26. [Kendrick FD](http://www.ncbi.nlm.nih.gov/pubmed/?term=Kendrick%20FD%5BAuthor%5D&cauthor=true&cauthor_uid=3037430), [Bonnin JM](http://www.ncbi.nlm.nih.gov/pubmed/?term=Bonnin%20JM%5BAuthor%5D&cauthor=true&cauthor_uid=3037430), [Garcia JH](http://www.ncbi.nlm.nih.gov/pubmed/?term=Garcia%20JH%5BAuthor%5D&cauthor=true&cauthor_uid=3037430). Metastases of a spinal glioblastoma multiforme into an intracranial arachnoid cyst. Neurosurgery. 1987 May;20(5):780-3. PMID: 3037430
27. [Kim WH](http://www.ncbi.nlm.nih.gov/pubmed/?term=Kim%20WH%5BAuthor%5D&cauthor=true&cauthor_uid=20526650), [Yoon SH](http://www.ncbi.nlm.nih.gov/pubmed/?term=Yoon%20SH%5BAuthor%5D&cauthor=true&cauthor_uid=20526650), [Kim CY](http://www.ncbi.nlm.nih.gov/pubmed/?term=Kim%20CY%5BAuthor%5D&cauthor=true&cauthor_uid=20526650), [Kim KJ](http://www.ncbi.nlm.nih.gov/pubmed/?term=Kim%20KJ%5BAuthor%5D&cauthor=true&cauthor_uid=20526650), [Lee MM](http://www.ncbi.nlm.nih.gov/pubmed/?term=Lee%20MM%5BAuthor%5D&cauthor=true&cauthor_uid=20526650), [Choe G](http://www.ncbi.nlm.nih.gov/pubmed/?term=Choe%20G%5BAuthor%5D&cauthor=true&cauthor_uid=20526650), [Kim IA](http://www.ncbi.nlm.nih.gov/pubmed/?term=Kim%20IA%5BAuthor%5D&cauthor=true&cauthor_uid=20526650), [Kim JH](http://www.ncbi.nlm.nih.gov/pubmed/?term=Kim%20JH%5BAuthor%5D&cauthor=true&cauthor_uid=20526650), [Kim YJ](http://www.ncbi.nlm.nih.gov/pubmed/?term=Kim%20YJ%5BAuthor%5D&cauthor=true&cauthor_uid=20526650), [Kim HJ](http://www.ncbi.nlm.nih.gov/pubmed/?term=Kim%20HJ%5BAuthor%5D&cauthor=true&cauthor_uid=20526650). Temozolomide for malignant primary spinal cord glioma: an experience of six cases and a literature review. J Neurooncol. 2011 Jan;101(2):247-54. PMID: 20526650
28. [Klase D](http://www.ncbi.nlm.nih.gov/pubmed/?term=Klase%20D%5BAuthor%5D&cauthor=true&cauthor_uid=17440736), [Gottschalk S](http://www.ncbi.nlm.nih.gov/pubmed/?term=Gottschalk%20S%5BAuthor%5D&cauthor=true&cauthor_uid=17440736), [Reusche E](http://www.ncbi.nlm.nih.gov/pubmed/?term=Reusche%20E%5BAuthor%5D&cauthor=true&cauthor_uid=17440736), [Hagel C](http://www.ncbi.nlm.nih.gov/pubmed/?term=Hagel%20C%5BAuthor%5D&cauthor=true&cauthor_uid=17440736), [Goebel E](http://www.ncbi.nlm.nih.gov/pubmed/?term=Goebel%20E%5BAuthor%5D&cauthor=true&cauthor_uid=17440736), [Tronnier V](http://www.ncbi.nlm.nih.gov/pubmed/?term=Tronnier%20V%5BAuthor%5D&cauthor=true&cauthor_uid=17440736), [Giese A](http://www.ncbi.nlm.nih.gov/pubmed/?term=Giese%20A%5BAuthor%5D&cauthor=true&cauthor_uid=17440736). Lumbosacral glioblastoma and leptomeningeal gliomatosis complicating the course of a cervicothoracic astrocytoma WHO grade II. Childs Nerv Syst. 2007 Aug;23(8):907-12. PMID: 17440736
29. [Klepstad P](http://www.ncbi.nlm.nih.gov/pubmed/?term=Klepstad%20P%5BAuthor%5D&cauthor=true&cauthor_uid=11902308), [Borchgrevink P](http://www.ncbi.nlm.nih.gov/pubmed/?term=Borchgrevink%20P%5BAuthor%5D&cauthor=true&cauthor_uid=11902308), [Hval B](http://www.ncbi.nlm.nih.gov/pubmed/?term=Hval%20B%5BAuthor%5D&cauthor=true&cauthor_uid=11902308), [Flaat S](http://www.ncbi.nlm.nih.gov/pubmed/?term=Flaat%20S%5BAuthor%5D&cauthor=true&cauthor_uid=11902308), [Kaasa S](http://www.ncbi.nlm.nih.gov/pubmed/?term=Kaasa%20S%5BAuthor%5D&cauthor=true&cauthor_uid=11902308). Long-term treatment with ketamine in a 12-year-old girl with severe neuropathic pain caused by a cervical spinal tumor. J Pediatr Hematol Oncol. 2001 Dec;23(9):616-9. PMID: 11902308
30. [König SA](http://www.ncbi.nlm.nih.gov/pubmed/?term=K%C3%B6nig%20SA%5BAuthor%5D&cauthor=true&cauthor_uid=21842458), [Roediger T](http://www.ncbi.nlm.nih.gov/pubmed/?term=Roediger%20T%5BAuthor%5D&cauthor=true&cauthor_uid=21842458), [Spetzger U](http://www.ncbi.nlm.nih.gov/pubmed/?term=Spetzger%20U%5BAuthor%5D&cauthor=true&cauthor_uid=21842458). Treatment of recurrent primary spinal glioblastoma multiforme--case report. J Neurol Surg A Cent Eur Neurosurg. 2012 Aug;73(4):256-61. PMID: 21842458
31. [Kubota T](http://www.ncbi.nlm.nih.gov/pubmed/?term=Kubota%20T%5BAuthor%5D&cauthor=true&cauthor_uid=3014673), [Kogure Y](http://www.ncbi.nlm.nih.gov/pubmed/?term=Kogure%20Y%5BAuthor%5D&cauthor=true&cauthor_uid=3014673), [Yamamoto S](http://www.ncbi.nlm.nih.gov/pubmed/?term=Yamamoto%20S%5BAuthor%5D&cauthor=true&cauthor_uid=3014673), [Matsubara S](http://www.ncbi.nlm.nih.gov/pubmed/?term=Matsubara%20S%5BAuthor%5D&cauthor=true&cauthor_uid=3014673), [Kitano T](http://www.ncbi.nlm.nih.gov/pubmed/?term=Kitano%20T%5BAuthor%5D&cauthor=true&cauthor_uid=3014673), [Hayashi M](http://www.ncbi.nlm.nih.gov/pubmed/?term=Hayashi%20M%5BAuthor%5D&cauthor=true&cauthor_uid=3014673). Calcification in glioblastoma multiforme of the cervical spinal cord. Surg Neurol. 1986 Aug;26(2):183-6. PMID: 3014673
32. [Kyoshima K](http://www.ncbi.nlm.nih.gov/pubmed/?term=Kyoshima%20K%5BAuthor%5D&cauthor=true&cauthor_uid=14642374), [Sakai K](http://www.ncbi.nlm.nih.gov/pubmed/?term=Sakai%20K%5BAuthor%5D&cauthor=true&cauthor_uid=14642374), [Goto T](http://www.ncbi.nlm.nih.gov/pubmed/?term=Goto%20T%5BAuthor%5D&cauthor=true&cauthor_uid=14642374), [Tanabe A](http://www.ncbi.nlm.nih.gov/pubmed/?term=Tanabe%20A%5BAuthor%5D&cauthor=true&cauthor_uid=14642374), [Sato A](http://www.ncbi.nlm.nih.gov/pubmed/?term=Sato%20A%5BAuthor%5D&cauthor=true&cauthor_uid=14642374), [Nagashima H](http://www.ncbi.nlm.nih.gov/pubmed/?term=Nagashima%20H%5BAuthor%5D&cauthor=true&cauthor_uid=14642374), [Nakayama J](http://www.ncbi.nlm.nih.gov/pubmed/?term=Nakayama%20J%5BAuthor%5D&cauthor=true&cauthor_uid=14642374). Gross total surgical removal of malignant glioma from the medulla oblongata: report of two adult cases with reference to surgical anatomy. J Clin Neurosci. 2004 Jan;11(1):75-80. PMID: 14642374
33. [Lau PK](http://www.ncbi.nlm.nih.gov/pubmed/?term=Lau%20PK%5BAuthor%5D&cauthor=true&cauthor_uid=24518393), [Lee G](http://www.ncbi.nlm.nih.gov/pubmed/?term=Lee%20G%5BAuthor%5D&cauthor=true&cauthor_uid=24518393), [Bynevelt M](http://www.ncbi.nlm.nih.gov/pubmed/?term=Bynevelt%20M%5BAuthor%5D&cauthor=true&cauthor_uid=24518393), [Nowak AK](http://www.ncbi.nlm.nih.gov/pubmed/?term=Nowak%20AK%5BAuthor%5D&cauthor=true&cauthor_uid=24518393). Marked functional improvement after combined chemoradiotherapy for cervical spine glioblastoma causing quadriparesis in an adolescent. BMJ Case Rep. 2014 Feb 11;2014. PMID: 24518393
34. Lasky J L, Moore, T, Liau, L. [Pediatric primary spinal glioblastoma with EWS gene rearrangement](http://apps.webofknowledge.com/full_record.do?product=UA&search_mode=GeneralSearch&qid=17&SID=Z1EHGmE2z8Plt6MyTIN&page=1&doc=1). NEURO-ONCOLOGY 2006 Oct; 8 (4): 468-468m. WOS:000240877301297
35. [Liu X](http://www.ncbi.nlm.nih.gov/pubmed/?term=Liu%20X%5BAuthor%5D&cauthor=true&cauthor_uid=20040012), [Germin BI](http://www.ncbi.nlm.nih.gov/pubmed/?term=Germin%20BI%5BAuthor%5D&cauthor=true&cauthor_uid=20040012), [Ekholm S](http://www.ncbi.nlm.nih.gov/pubmed/?term=Ekholm%20S%5BAuthor%5D&cauthor=true&cauthor_uid=20040012). A case of cervical spinal cord glioblastoma diagnosed with MR diffusion tensor and perfusion imaging. J Neuroimaging. 2011 Jul;21(3):292-6. PMID: 20040012
36. [Lober R](http://www.ncbi.nlm.nih.gov/pubmed/?term=Lober%20R%5BAuthor%5D&cauthor=true&cauthor_uid=21139963), [Sharma S](http://www.ncbi.nlm.nih.gov/pubmed/?term=Sharma%20S%5BAuthor%5D&cauthor=true&cauthor_uid=21139963), [Bell B](http://www.ncbi.nlm.nih.gov/pubmed/?term=Bell%20B%5BAuthor%5D&cauthor=true&cauthor_uid=21139963), [Free A](http://www.ncbi.nlm.nih.gov/pubmed/?term=Free%20A%5BAuthor%5D&cauthor=true&cauthor_uid=21139963), [Figueroa R](http://www.ncbi.nlm.nih.gov/pubmed/?term=Figueroa%20R%5BAuthor%5D&cauthor=true&cauthor_uid=21139963), [Sheils CW](http://www.ncbi.nlm.nih.gov/pubmed/?term=Sheils%20CW%5BAuthor%5D&cauthor=true&cauthor_uid=21139963), [Lee M](http://www.ncbi.nlm.nih.gov/pubmed/?term=Lee%20M%5BAuthor%5D&cauthor=true&cauthor_uid=21139963), [Cowell J](http://www.ncbi.nlm.nih.gov/pubmed/?term=Cowell%20J%5BAuthor%5D&cauthor=true&cauthor_uid=21139963). Pediatric primary intramedullary spinal cord glioblastoma. Rare Tumors. 2010 Sep 30;2(3):e48. PMID: 21139963
37. [Marchan EM](http://www.ncbi.nlm.nih.gov/pubmed/?term=Marchan%20EM%5BAuthor%5D&cauthor=true&cauthor_uid=18074692), [Sekula RF Jr](http://www.ncbi.nlm.nih.gov/pubmed/?term=Sekula%20RF%20Jr%5BAuthor%5D&cauthor=true&cauthor_uid=18074692), [Jannetta PJ](http://www.ncbi.nlm.nih.gov/pubmed/?term=Jannetta%20PJ%5BAuthor%5D&cauthor=true&cauthor_uid=18074692), [Quigley MR](http://www.ncbi.nlm.nih.gov/pubmed/?term=Quigley%20MR%5BAuthor%5D&cauthor=true&cauthor_uid=18074692). Long-term survival enhanced by cordectomy in a patient with a spinal glioblastoma multiforme and paraplegia. Case report. J Neurosurg Spine. 2007 Dec;7(6):656-9. PMID: 18074692
38. [Matsumoto T](http://www.ncbi.nlm.nih.gov/pubmed/?term=Matsumoto%20T%5BAuthor%5D&cauthor=true&cauthor_uid=19086699), [Urasaki E](http://www.ncbi.nlm.nih.gov/pubmed/?term=Urasaki%20E%5BAuthor%5D&cauthor=true&cauthor_uid=19086699), [Soejima Y](http://www.ncbi.nlm.nih.gov/pubmed/?term=Soejima%20Y%5BAuthor%5D&cauthor=true&cauthor_uid=19086699), [Nakano Y](http://www.ncbi.nlm.nih.gov/pubmed/?term=Nakano%20Y%5BAuthor%5D&cauthor=true&cauthor_uid=19086699), [Yokota A](http://www.ncbi.nlm.nih.gov/pubmed/?term=Yokota%20A%5BAuthor%5D&cauthor=true&cauthor_uid=19086699), [Nishizawa S](http://www.ncbi.nlm.nih.gov/pubmed/?term=Nishizawa%20S%5BAuthor%5D&cauthor=true&cauthor_uid=19086699). Cervical intramedullary glioblastoma: report of a long-term survival case and a review of the literature. J UOEH. 2008 Dec 1;30(4):413-20. PMID: 19086699
39. [Mayer RR](http://www.ncbi.nlm.nih.gov/pubmed/?term=Mayer%20RR%5BAuthor%5D&cauthor=true&cauthor_uid=22040726), [Warmouth GM](http://www.ncbi.nlm.nih.gov/pubmed/?term=Warmouth%20GM%5BAuthor%5D&cauthor=true&cauthor_uid=22040726), [Troxell M](http://www.ncbi.nlm.nih.gov/pubmed/?term=Troxell%20M%5BAuthor%5D&cauthor=true&cauthor_uid=22040726), [Adesina AM](http://www.ncbi.nlm.nih.gov/pubmed/?term=Adesina%20AM%5BAuthor%5D&cauthor=true&cauthor_uid=22040726), [Kass JS](http://www.ncbi.nlm.nih.gov/pubmed/?term=Kass%20JS%5BAuthor%5D&cauthor=true&cauthor_uid=22040726). Glioblastoma multiforme of the conus medullaris in a 28-year-old female: a case report and review of the literature. Clin Neurol Neurosurg. 2012 Apr;114(3):275-7. PMID: 22040726
40. [Medhkour A](http://www.ncbi.nlm.nih.gov/pubmed/?term=Medhkour%20A%5BAuthor%5D&cauthor=true&cauthor_uid=15936395), [Chan M](http://www.ncbi.nlm.nih.gov/pubmed/?term=Chan%20M%5BAuthor%5D&cauthor=true&cauthor_uid=15936395). Extremely rare glioblastoma multiforme of the conus medullaris with holocord and brain stem metastases, leading to cranial nerve deficit and respiratory failure: a case report and review of the literature. Surg Neurol. 2005 Jun;63(6):576-82; discussion 582-3.PMID: 15936395
41. [Merchant TE](http://www.ncbi.nlm.nih.gov/pubmed/?term=Merchant%20TE%5BAuthor%5D&cauthor=true&cauthor_uid=10202299), [Nguyen D](http://www.ncbi.nlm.nih.gov/pubmed/?term=Nguyen%20D%5BAuthor%5D&cauthor=true&cauthor_uid=10202299), [Thompson SJ](http://www.ncbi.nlm.nih.gov/pubmed/?term=Thompson%20SJ%5BAuthor%5D&cauthor=true&cauthor_uid=10202299), [Reardon DA](http://www.ncbi.nlm.nih.gov/pubmed/?term=Reardon%20DA%5BAuthor%5D&cauthor=true&cauthor_uid=10202299), [Kun LE](http://www.ncbi.nlm.nih.gov/pubmed/?term=Kun%20LE%5BAuthor%5D&cauthor=true&cauthor_uid=10202299), [Sanford RA](http://www.ncbi.nlm.nih.gov/pubmed/?term=Sanford%20RA%5BAuthor%5D&cauthor=true&cauthor_uid=10202299). High-grade pediatric spinal cord tumors. Pediatr Neurosurg. 1999 Jan;30(1):1-5. PMID: 10202299
42. [Morais N](http://www.ncbi.nlm.nih.gov/pubmed/?term=Morais%20N%5BAuthor%5D&cauthor=true&cauthor_uid=23426283), [Mascarenhas L](http://www.ncbi.nlm.nih.gov/pubmed/?term=Mascarenhas%20L%5BAuthor%5D&cauthor=true&cauthor_uid=23426283), [Soares-Fernandes JP](http://www.ncbi.nlm.nih.gov/pubmed/?term=Soares-Fernandes%20JP%5BAuthor%5D&cauthor=true&cauthor_uid=23426283), [Silva A](http://www.ncbi.nlm.nih.gov/pubmed/?term=Silva%20A%5BAuthor%5D&cauthor=true&cauthor_uid=23426283), [Magalhães Z](http://www.ncbi.nlm.nih.gov/pubmed/?term=Magalh%C3%A3es%20Z%5BAuthor%5D&cauthor=true&cauthor_uid=23426283), [Costa JA](http://www.ncbi.nlm.nih.gov/pubmed/?term=Costa%20JA%5BAuthor%5D&cauthor=true&cauthor_uid=23426283). Primary spinal glioblastoma: A case report and review of the literature. Oncol Lett. 2013 Mar;5(3):992-996. PMID: 23426283
43. [Mori K](http://www.ncbi.nlm.nih.gov/pubmed/?term=Mori%20K%5BAuthor%5D&cauthor=true&cauthor_uid=22197783), [Imai S](http://www.ncbi.nlm.nih.gov/pubmed/?term=Imai%20S%5BAuthor%5D&cauthor=true&cauthor_uid=22197783), [Shimizu J](http://www.ncbi.nlm.nih.gov/pubmed/?term=Shimizu%20J%5BAuthor%5D&cauthor=true&cauthor_uid=22197783), [Taga T](http://www.ncbi.nlm.nih.gov/pubmed/?term=Taga%20T%5BAuthor%5D&cauthor=true&cauthor_uid=22197783), [Ishida M](http://www.ncbi.nlm.nih.gov/pubmed/?term=Ishida%20M%5BAuthor%5D&cauthor=true&cauthor_uid=22197783), [Matsusue Y](http://www.ncbi.nlm.nih.gov/pubmed/?term=Matsusue%20Y%5BAuthor%5D&cauthor=true&cauthor_uid=22197783). Spinal glioblastoma multiforme of the conus medullaris with holocordal and intracranial spread in a child: a case report and review of the literature. Spine J. 2012 Jan;12(1):e1-6. PMID: 22197783
44. [Mortara R](http://www.ncbi.nlm.nih.gov/pubmed/?term=Mortara%20R%5BAuthor%5D&cauthor=true&cauthor_uid=4362014), [Parker JC Jr](http://www.ncbi.nlm.nih.gov/pubmed/?term=Parker%20JC%20Jr%5BAuthor%5D&cauthor=true&cauthor_uid=4362014), [Brooks WH](http://www.ncbi.nlm.nih.gov/pubmed/?term=Brooks%20WH%5BAuthor%5D&cauthor=true&cauthor_uid=4362014). Glioblastoma multiforme of the spinal cord. Surg Neurol. 1974 Mar;2(2):115-9. PMID: 4362014
45. [Newman RP](http://www.ncbi.nlm.nih.gov/pubmed/?term=Newman%20RP%5BAuthor%5D&cauthor=true&cauthor_uid=6326713), [Schaefer EJ](http://www.ncbi.nlm.nih.gov/pubmed/?term=Schaefer%20EJ%5BAuthor%5D&cauthor=true&cauthor_uid=6326713), [Thomas CB](http://www.ncbi.nlm.nih.gov/pubmed/?term=Thomas%20CB%5BAuthor%5D&cauthor=true&cauthor_uid=6326713), [Oldfield EH](http://www.ncbi.nlm.nih.gov/pubmed/?term=Oldfield%20EH%5BAuthor%5D&cauthor=true&cauthor_uid=6326713). Abetalipoproteinemia and metastatic spinal cord glioblastoma. Arch Neurol. 1984 May;41(5):554-6. PMID: 6326713
46. [Ng C](http://www.ncbi.nlm.nih.gov/pubmed/?term=Ng%20C%5BAuthor%5D&cauthor=true&cauthor_uid=17436928), [Fairhall J](http://www.ncbi.nlm.nih.gov/pubmed/?term=Fairhall%20J%5BAuthor%5D&cauthor=true&cauthor_uid=17436928), [Rathmalgoda C](http://www.ncbi.nlm.nih.gov/pubmed/?term=Rathmalgoda%20C%5BAuthor%5D&cauthor=true&cauthor_uid=17436928), [Stening W](http://www.ncbi.nlm.nih.gov/pubmed/?term=Stening%20W%5BAuthor%5D&cauthor=true&cauthor_uid=17436928), [Smee R](http://www.ncbi.nlm.nih.gov/pubmed/?term=Smee%20R%5BAuthor%5D&cauthor=true&cauthor_uid=17436928). Spinal cord glioblastoma multiforme induced by radiation after treatment for Hodgkin disease. Case report. J Neurosurg Spine. 2007 Apr;6(4):364-7. PMID: 17436928
47. [Nishio S](http://www.ncbi.nlm.nih.gov/pubmed/?term=Nishio%20S%5BAuthor%5D&cauthor=true&cauthor_uid=10847645), [Morioka T](http://www.ncbi.nlm.nih.gov/pubmed/?term=Morioka%20T%5BAuthor%5D&cauthor=true&cauthor_uid=10847645), [Fujii K](http://www.ncbi.nlm.nih.gov/pubmed/?term=Fujii%20K%5BAuthor%5D&cauthor=true&cauthor_uid=10847645), [Inamura T](http://www.ncbi.nlm.nih.gov/pubmed/?term=Inamura%20T%5BAuthor%5D&cauthor=true&cauthor_uid=10847645), [Fukui M](http://www.ncbi.nlm.nih.gov/pubmed/?term=Fukui%20M%5BAuthor%5D&cauthor=true&cauthor_uid=10847645). Spinal cord gliomas: management and outcome with reference to adjuvant therapy. J Clin Neurosci. 2000 Jan;7(1):20-3. PMID: 10847645
48. O'Connell JEA. The subarachnoidal dissemination of spinal tumours. J Neurol Neurosurg Psychiat 1946; 9: 55-62
49. [O'Halloran PJ](http://www.ncbi.nlm.nih.gov/pubmed/?term=O'Halloran%20PJ%5BAuthor%5D&cauthor=true&cauthor_uid=23319103), [Farrell M](http://www.ncbi.nlm.nih.gov/pubmed/?term=Farrell%20M%5BAuthor%5D&cauthor=true&cauthor_uid=23319103), [Caird J](http://www.ncbi.nlm.nih.gov/pubmed/?term=Caird%20J%5BAuthor%5D&cauthor=true&cauthor_uid=23319103), [Capra M](http://www.ncbi.nlm.nih.gov/pubmed/?term=Capra%20M%5BAuthor%5D&cauthor=true&cauthor_uid=23319103), [O'Brien D](http://www.ncbi.nlm.nih.gov/pubmed/?term=O'Brien%20D%5BAuthor%5D&cauthor=true&cauthor_uid=23319103). Paediatric spinal glioblastoma: case report and review of therapeutic strategies. Childs Nerv Syst. 2013 Mar;29(3):367-74. PMID: 23319103
50. [Oake C](http://www.ncbi.nlm.nih.gov/pubmed/?term=Oake%20C%5BAuthor%5D&cauthor=true&cauthor_uid=16884424), [Borg MF](http://www.ncbi.nlm.nih.gov/pubmed/?term=Borg%20MF%5BAuthor%5D&cauthor=true&cauthor_uid=16884424), [Hanieh A](http://www.ncbi.nlm.nih.gov/pubmed/?term=Hanieh%20A%5BAuthor%5D&cauthor=true&cauthor_uid=16884424), [Byard RW](http://www.ncbi.nlm.nih.gov/pubmed/?term=Byard%20RW%5BAuthor%5D&cauthor=true&cauthor_uid=16884424). Childhood glioblastoma multiforme of the spinal cord. Australas Radiol. 2006 Aug;50(4):360-3. PMID: 16884424
51. [Ononiwu C](http://www.ncbi.nlm.nih.gov/pubmed/?term=Ononiwu%20C%5BAuthor%5D&cauthor=true&cauthor_uid=22307824), [Mehta V](http://www.ncbi.nlm.nih.gov/pubmed/?term=Mehta%20V%5BAuthor%5D&cauthor=true&cauthor_uid=22307824), [Bettegowda C](http://www.ncbi.nlm.nih.gov/pubmed/?term=Bettegowda%20C%5BAuthor%5D&cauthor=true&cauthor_uid=22307824), [Jallo G](http://www.ncbi.nlm.nih.gov/pubmed/?term=Jallo%20G%5BAuthor%5D&cauthor=true&cauthor_uid=22307824). Pediatric spinal glioblastoma multiforme: current treatment strategies and possible predictors of survival. Childs Nerv Syst. 2012 May;28(5):715-20. PMID: 22307824
52. [Ozgiray E](http://www.ncbi.nlm.nih.gov/pubmed/?term=Ozgiray%20E%5BAuthor%5D&cauthor=true&cauthor_uid=24310474), [Akay A](http://www.ncbi.nlm.nih.gov/pubmed/?term=Akay%20A%5BAuthor%5D&cauthor=true&cauthor_uid=24310474), [Ertan Y](http://www.ncbi.nlm.nih.gov/pubmed/?term=Ertan%20Y%5BAuthor%5D&cauthor=true&cauthor_uid=24310474), [Cagli S](http://www.ncbi.nlm.nih.gov/pubmed/?term=Cagli%20S%5BAuthor%5D&cauthor=true&cauthor_uid=24310474), [Oktar N](http://www.ncbi.nlm.nih.gov/pubmed/?term=Oktar%20N%5BAuthor%5D&cauthor=true&cauthor_uid=24310474), [Ozdamar N](http://www.ncbi.nlm.nih.gov/pubmed/?term=Ozdamar%20N%5BAuthor%5D&cauthor=true&cauthor_uid=24310474). Primary glioblastoma of the medulla spinalis: a report of three cases and review of the literature. Turk Neurosurg. 2013;23(6):828-34. PMID: 24310474
53. [Paraskevopoulos D](http://www.ncbi.nlm.nih.gov/pubmed/?term=Paraskevopoulos%20D%5BAuthor%5D&cauthor=true&cauthor_uid=20461521), [Patsalas I](http://www.ncbi.nlm.nih.gov/pubmed/?term=Patsalas%20I%5BAuthor%5D&cauthor=true&cauthor_uid=20461521), [Karkavelas G](http://www.ncbi.nlm.nih.gov/pubmed/?term=Karkavelas%20G%5BAuthor%5D&cauthor=true&cauthor_uid=20461521), [Foroglou N](http://www.ncbi.nlm.nih.gov/pubmed/?term=Foroglou%20N%5BAuthor%5D&cauthor=true&cauthor_uid=20461521), [Magras I](http://www.ncbi.nlm.nih.gov/pubmed/?term=Magras%20I%5BAuthor%5D&cauthor=true&cauthor_uid=20461521), [Selviaridis P](http://www.ncbi.nlm.nih.gov/pubmed/?term=Selviaridis%20P%5BAuthor%5D&cauthor=true&cauthor_uid=20461521). Pilomyxoid astrocytoma of the cervical spinal cord in a child with rapid progression into glioblastoma: case report and literature review. Childs Nerv Syst. 2011 Feb;27(2):313-21. PMID: 20461521
54. Prasad G L, Borkar SA, Subbarao KC, Suri V, Mahapatra AK. Primary spinal cord glioblastoma multiforme: A report of two cases. Neurol India 2012;60:333-5. **DOI**: 10.4103/0028-3886.98530
55. [Przybylski GJ](http://www.ncbi.nlm.nih.gov/pubmed/?term=Przybylski%20GJ%5BAuthor%5D&cauthor=true&cauthor_uid=9298273), [Albright AL](http://www.ncbi.nlm.nih.gov/pubmed/?term=Albright%20AL%5BAuthor%5D&cauthor=true&cauthor_uid=9298273), [Martinez AJ](http://www.ncbi.nlm.nih.gov/pubmed/?term=Martinez%20AJ%5BAuthor%5D&cauthor=true&cauthor_uid=9298273). Spinal cord astrocytomas: long-term results comparing treatments in children. Childs Nerv Syst. 1997 Jul;13(7):375-82. PMID: 9298273
56. [Queiroz Lde S](http://www.ncbi.nlm.nih.gov/pubmed/?term=Queiroz%20Lde%20S%5BAuthor%5D&cauthor=true&cauthor_uid=4374862), [da Cruz Neto JN](http://www.ncbi.nlm.nih.gov/pubmed/?term=da%20Cruz%20Neto%20JN%5BAuthor%5D&cauthor=true&cauthor_uid=4374862), [Lopes de Faria J](http://www.ncbi.nlm.nih.gov/pubmed/?term=Lopes%20de%20Faria%20J%5BAuthor%5D&cauthor=true&cauthor_uid=4374862). Glioblastoma multiforme of the medulla oblangata. Acta Neuropathol. 1974;29(4):355-60. PMID: 4374862
57. Rand, R. W., & Rand, C. W. Intraspinal tumors of childhood. Blackwell. 1960
58. [Randakevičienė G](http://www.ncbi.nlm.nih.gov/pubmed/?term=Randakevi%C4%8Dien%C4%97%20G%5BAuthor%5D&cauthor=true&cauthor_uid=24247921), [Gleiznienė R](http://www.ncbi.nlm.nih.gov/pubmed/?term=Gleiznien%C4%97%20R%5BAuthor%5D&cauthor=true&cauthor_uid=24247921), [Basevičius A](http://www.ncbi.nlm.nih.gov/pubmed/?term=Basevi%C4%8Dius%20A%5BAuthor%5D&cauthor=true&cauthor_uid=24247921), [Lukoševičius S](http://www.ncbi.nlm.nih.gov/pubmed/?term=Luko%C5%A1evi%C4%8Dius%20S%5BAuthor%5D&cauthor=true&cauthor_uid=24247921). An extremely rare case of glioblastoma multiforme of the spinal cord. Medicina (Kaunas). 2013;49(5):242-5. PMID: 24247921
59. [Roessmann U](http://www.ncbi.nlm.nih.gov/pubmed/?term=Roessmann%20U%5BAuthor%5D&cauthor=true&cauthor_uid=4339732), [Weiss MH](http://www.ncbi.nlm.nih.gov/pubmed/?term=Weiss%20MH%5BAuthor%5D&cauthor=true&cauthor_uid=4339732). Giganto-cellular glioblastoma of the lumbosacral spinal cord. Acta Neuropathol. 1972;20(3):264-6. PMID: 4339732
60. [Rokes CA](http://www.ncbi.nlm.nih.gov/pubmed/?term=Rokes%20CA%5BAuthor%5D&cauthor=true&cauthor_uid=20463608), [Remke M](http://www.ncbi.nlm.nih.gov/pubmed/?term=Remke%20M%5BAuthor%5D&cauthor=true&cauthor_uid=20463608), [Guha-Thakurta N](http://www.ncbi.nlm.nih.gov/pubmed/?term=Guha-Thakurta%20N%5BAuthor%5D&cauthor=true&cauthor_uid=20463608), [Witt O](http://www.ncbi.nlm.nih.gov/pubmed/?term=Witt%20O%5BAuthor%5D&cauthor=true&cauthor_uid=20463608), [Korshunov A](http://www.ncbi.nlm.nih.gov/pubmed/?term=Korshunov%20A%5BAuthor%5D&cauthor=true&cauthor_uid=20463608), [Pfister S](http://www.ncbi.nlm.nih.gov/pubmed/?term=Pfister%20S%5BAuthor%5D&cauthor=true&cauthor_uid=20463608), [Wolff JE](http://www.ncbi.nlm.nih.gov/pubmed/?term=Wolff%20JE%5BAuthor%5D&cauthor=true&cauthor_uid=20463608). Sorafenib plus valproic acid for infant spinal glioblastoma. J Pediatr Hematol Oncol. 2010 Aug;32(6):511-4. PMID: 20463608
61. [Rossberg C](http://www.ncbi.nlm.nih.gov/pubmed/?term=Rossberg%20C%5BAuthor%5D&cauthor=true&cauthor_uid=2841617), [Litzenberger J](http://www.ncbi.nlm.nih.gov/pubmed/?term=Litzenberger%20J%5BAuthor%5D&cauthor=true&cauthor_uid=2841617). Intramedullary glioblastoma multiforme with unusual, intracranial neoplastic meningeosis. Nervenarzt. 1988 Jul;59(7):401-4. PMID: 2841617
62. [Salem A](http://www.ncbi.nlm.nih.gov/pubmed/?term=Salem%20A%5BAuthor%5D&cauthor=true&cauthor_uid=23886673), [Alshorbaji A](http://www.ncbi.nlm.nih.gov/pubmed/?term=Alshorbaji%20A%5BAuthor%5D&cauthor=true&cauthor_uid=23886673), [Almousa A](http://www.ncbi.nlm.nih.gov/pubmed/?term=Almousa%20A%5BAuthor%5D&cauthor=true&cauthor_uid=23886673). Spinal glioblastoma multiforme. Hematol Oncol Stem Cell Ther. 2014 Mar;7(1):56-7. PMID: 23886673
63. [Sanborn MR](http://www.ncbi.nlm.nih.gov/pubmed/?term=Sanborn%20MR%5BAuthor%5D&cauthor=true&cauthor_uid=21435883), [Pramick M](http://www.ncbi.nlm.nih.gov/pubmed/?term=Pramick%20M%5BAuthor%5D&cauthor=true&cauthor_uid=21435883), [Brooks J](http://www.ncbi.nlm.nih.gov/pubmed/?term=Brooks%20J%5BAuthor%5D&cauthor=true&cauthor_uid=21435883), [Welch WC](http://www.ncbi.nlm.nih.gov/pubmed/?term=Welch%20WC%5BAuthor%5D&cauthor=true&cauthor_uid=21435883). Glioblastoma multiforme in the adult conus medullaris. J Clin Neurosci. 2011 Jun;18(6):842-3. PMID: 21435883
64. [Santi M](http://www.ncbi.nlm.nih.gov/pubmed/?term=Santi%20M%5BAuthor%5D&cauthor=true&cauthor_uid=12879473), [Mena H](http://www.ncbi.nlm.nih.gov/pubmed/?term=Mena%20H%5BAuthor%5D&cauthor=true&cauthor_uid=12879473), [Wong K](http://www.ncbi.nlm.nih.gov/pubmed/?term=Wong%20K%5BAuthor%5D&cauthor=true&cauthor_uid=12879473), [Koeller K](http://www.ncbi.nlm.nih.gov/pubmed/?term=Koeller%20K%5BAuthor%5D&cauthor=true&cauthor_uid=12879473), [Olsen C](http://www.ncbi.nlm.nih.gov/pubmed/?term=Olsen%20C%5BAuthor%5D&cauthor=true&cauthor_uid=12879473), [Rushing EJ](http://www.ncbi.nlm.nih.gov/pubmed/?term=Rushing%20EJ%5BAuthor%5D&cauthor=true&cauthor_uid=12879473). Spinal cord malignant astrocytomas. Clinicopathologic features in 36 cases. Cancer. 2003 Aug 1;98(3):554-61. PMID: 12879473
65. [Scarrow AM](http://www.ncbi.nlm.nih.gov/pubmed/?term=Scarrow%20AM%5BAuthor%5D&cauthor=true&cauthor_uid=10996716), [Rajendran P](http://www.ncbi.nlm.nih.gov/pubmed/?term=Rajendran%20P%5BAuthor%5D&cauthor=true&cauthor_uid=10996716), [Welch WC](http://www.ncbi.nlm.nih.gov/pubmed/?term=Welch%20WC%5BAuthor%5D&cauthor=true&cauthor_uid=10996716). Glioblastoma multiforme of the conus medullaris. Clin Neurol Neurosurg. 2000 Sep;102(3):166-7. PMID: 10996716
66. [Shirato H](http://www.ncbi.nlm.nih.gov/pubmed/?term=Shirato%20H%5BAuthor%5D&cauthor=true&cauthor_uid=7673019), [Kamada T](http://www.ncbi.nlm.nih.gov/pubmed/?term=Kamada%20T%5BAuthor%5D&cauthor=true&cauthor_uid=7673019), [Hida K](http://www.ncbi.nlm.nih.gov/pubmed/?term=Hida%20K%5BAuthor%5D&cauthor=true&cauthor_uid=7673019), [Koyanagi I](http://www.ncbi.nlm.nih.gov/pubmed/?term=Koyanagi%20I%5BAuthor%5D&cauthor=true&cauthor_uid=7673019), [Iwasaki Y](http://www.ncbi.nlm.nih.gov/pubmed/?term=Iwasaki%20Y%5BAuthor%5D&cauthor=true&cauthor_uid=7673019), [Miyasaka K](http://www.ncbi.nlm.nih.gov/pubmed/?term=Miyasaka%20K%5BAuthor%5D&cauthor=true&cauthor_uid=7673019), [Abe H](http://www.ncbi.nlm.nih.gov/pubmed/?term=Abe%20H%5BAuthor%5D&cauthor=true&cauthor_uid=7673019). The role of radiotherapy in the management of spinal cord glioma. Int J Radiat Oncol Biol Phys. 1995 Sep 30;33(2):323-8. PMID: 7673019
67. [Shuangshoti S](http://www.ncbi.nlm.nih.gov/pubmed/?term=Shuangshoti%20S%5BAuthor%5D&cauthor=true&cauthor_uid=8855617), [Shuangshoti S](http://www.ncbi.nlm.nih.gov/pubmed/?term=Shuangshoti%20S%5BAuthor%5D&cauthor=true&cauthor_uid=8855617). Primary diffuse leptomeningeal glioblastoma multiforme of brainstem and spinal cord clinically mimicking meningitis: case report and review of literature. J Med Assoc Thai. 1996 Jun;79(6):403-8. PMID: 8855617
68. [Singh PK](http://www.ncbi.nlm.nih.gov/pubmed/?term=Singh%20PK%5BAuthor%5D&cauthor=true&cauthor_uid=20025156), [Singh VK](http://www.ncbi.nlm.nih.gov/pubmed/?term=Singh%20VK%5BAuthor%5D&cauthor=true&cauthor_uid=20025156), [Tomar J](http://www.ncbi.nlm.nih.gov/pubmed/?term=Tomar%20J%5BAuthor%5D&cauthor=true&cauthor_uid=20025156), [Azam A](http://www.ncbi.nlm.nih.gov/pubmed/?term=Azam%20A%5BAuthor%5D&cauthor=true&cauthor_uid=20025156), [Gupta S](http://www.ncbi.nlm.nih.gov/pubmed/?term=Gupta%20S%5BAuthor%5D&cauthor=true&cauthor_uid=20025156), [Kumar S](http://www.ncbi.nlm.nih.gov/pubmed/?term=Kumar%20S%5BAuthor%5D&cauthor=true&cauthor_uid=20025156). Spinal glioblastoma multiforme: unusual cause of post-traumatic tetraparesis. J Spinal Cord Med. 2009;32(5):583-6. PMID: 20025156
69. Sloof JL, Kernohan JW, MacCarty CS. Primary Intramedullary Tumors of the Spinal Cord and Filum Terminale. Philadelphia: W. B. Saunders, 1964; 31-61.
70. [Stecco A](http://www.ncbi.nlm.nih.gov/pubmed/?term=Stecco%20A%5BAuthor%5D&cauthor=true&cauthor_uid=16155176), [Quirico C](http://www.ncbi.nlm.nih.gov/pubmed/?term=Quirico%20C%5BAuthor%5D&cauthor=true&cauthor_uid=16155176), [Giampietro A](http://www.ncbi.nlm.nih.gov/pubmed/?term=Giampietro%20A%5BAuthor%5D&cauthor=true&cauthor_uid=16155176), [Sessa G](http://www.ncbi.nlm.nih.gov/pubmed/?term=Sessa%20G%5BAuthor%5D&cauthor=true&cauthor_uid=16155176), [Boldorini R](http://www.ncbi.nlm.nih.gov/pubmed/?term=Boldorini%20R%5BAuthor%5D&cauthor=true&cauthor_uid=16155176), [Carriero A](http://www.ncbi.nlm.nih.gov/pubmed/?term=Carriero%20A%5BAuthor%5D&cauthor=true&cauthor_uid=16155176). Glioblastoma multiforme of the conus medullaris in a child: description of a case and literature review. AJNR Am J Neuroradiol. 2005 Sep;26(8):2157-60. PMID: 16155176
71. [Strik HM](http://www.ncbi.nlm.nih.gov/pubmed/?term=Strik%20HM%5BAuthor%5D&cauthor=true&cauthor_uid=11263503), [Effenberger O](http://www.ncbi.nlm.nih.gov/pubmed/?term=Effenberger%20O%5BAuthor%5D&cauthor=true&cauthor_uid=11263503), [Schäfer O](http://www.ncbi.nlm.nih.gov/pubmed/?term=Sch%C3%A4fer%20O%5BAuthor%5D&cauthor=true&cauthor_uid=11263503), [Risch U](http://www.ncbi.nlm.nih.gov/pubmed/?term=Risch%20U%5BAuthor%5D&cauthor=true&cauthor_uid=11263503), [Wickboldt J](http://www.ncbi.nlm.nih.gov/pubmed/?term=Wickboldt%20J%5BAuthor%5D&cauthor=true&cauthor_uid=11263503), [Meyermann R](http://www.ncbi.nlm.nih.gov/pubmed/?term=Meyermann%20R%5BAuthor%5D&cauthor=true&cauthor_uid=11263503). A case of spinal glioblastoma multiforme: immunohistochemical study and review of the literature. J Neurooncol. 2000 Dec;50(3):239-43. PMID: 11263503
72. [Tendulkar RD](http://www.ncbi.nlm.nih.gov/pubmed/?term=Tendulkar%20RD%5BAuthor%5D&cauthor=true&cauthor_uid=20346593), [Pai Panandiker AS](http://www.ncbi.nlm.nih.gov/pubmed/?term=Pai%20Panandiker%20AS%5BAuthor%5D&cauthor=true&cauthor_uid=20346593), [Wu S](http://www.ncbi.nlm.nih.gov/pubmed/?term=Wu%20S%5BAuthor%5D&cauthor=true&cauthor_uid=20346593), [Kun LE](http://www.ncbi.nlm.nih.gov/pubmed/?term=Kun%20LE%5BAuthor%5D&cauthor=true&cauthor_uid=20346593), [Broniscer A](http://www.ncbi.nlm.nih.gov/pubmed/?term=Broniscer%20A%5BAuthor%5D&cauthor=true&cauthor_uid=20346593), [Sanford RA](http://www.ncbi.nlm.nih.gov/pubmed/?term=Sanford%20RA%5BAuthor%5D&cauthor=true&cauthor_uid=20346593), [Merchant TE](http://www.ncbi.nlm.nih.gov/pubmed/?term=Merchant%20TE%5BAuthor%5D&cauthor=true&cauthor_uid=20346593). Irradiation of pediatric high-grade spinal cord tumors. Int J Radiat Oncol Biol Phys. 2010 Dec 1;78(5):1451-6. PMID: 20346593
73. [Tseng HM](http://www.ncbi.nlm.nih.gov/pubmed/?term=Tseng%20HM%5BAuthor%5D&cauthor=true&cauthor_uid=20838208), [Kuo LT](http://www.ncbi.nlm.nih.gov/pubmed/?term=Kuo%20LT%5BAuthor%5D&cauthor=true&cauthor_uid=20838208), [Lien HC](http://www.ncbi.nlm.nih.gov/pubmed/?term=Lien%20HC%5BAuthor%5D&cauthor=true&cauthor_uid=20838208), [Liu KL](http://www.ncbi.nlm.nih.gov/pubmed/?term=Liu%20KL%5BAuthor%5D&cauthor=true&cauthor_uid=20838208), [Liu MT](http://www.ncbi.nlm.nih.gov/pubmed/?term=Liu%20MT%5BAuthor%5D&cauthor=true&cauthor_uid=20838208), [Huang CY](http://www.ncbi.nlm.nih.gov/pubmed/?term=Huang%20CY%5BAuthor%5D&cauthor=true&cauthor_uid=20838208). Prolonged survival of a patient with cervical intramedullary glioblastoma multiforme treated with total resection, radiation therapy, and temozolomide. Anticancer Drugs. 2010 Nov;21(10):963-7. PMID: 20838208
74. [Varghese SS](http://www.ncbi.nlm.nih.gov/pubmed/?term=Varghese%20SS%5BAuthor%5D&cauthor=true&cauthor_uid=24959488), [Sebastian P](http://www.ncbi.nlm.nih.gov/pubmed/?term=Sebastian%20P%5BAuthor%5D&cauthor=true&cauthor_uid=24959488)1, [Joseph V](http://www.ncbi.nlm.nih.gov/pubmed/?term=Joseph%20V%5BAuthor%5D&cauthor=true&cauthor_uid=24959488), [Chacko G](http://www.ncbi.nlm.nih.gov/pubmed/?term=Chacko%20G%5BAuthor%5D&cauthor=true&cauthor_uid=24959488), [Backianathan S](http://www.ncbi.nlm.nih.gov/pubmed/?term=Backianathan%20S%5BAuthor%5D&cauthor=true&cauthor_uid=24959488). An unusually long survival of a patient with glioblastoma of spinal cord: a case report. J Clin Diagn Res. 2014 Apr;8(4):QD01-3. PMID: 24959488
75. [Viljoen S](http://www.ncbi.nlm.nih.gov/pubmed/?term=Viljoen%20S%5BAuthor%5D&cauthor=true&cauthor_uid=25101196), [Hitchon PW](http://www.ncbi.nlm.nih.gov/pubmed/?term=Hitchon%20PW%5BAuthor%5D&cauthor=true&cauthor_uid=25101196), [Ahmed R](http://www.ncbi.nlm.nih.gov/pubmed/?term=Ahmed%20R%5BAuthor%5D&cauthor=true&cauthor_uid=25101196), [Kirby PA](http://www.ncbi.nlm.nih.gov/pubmed/?term=Kirby%20PA%5BAuthor%5D&cauthor=true&cauthor_uid=25101196). Cordectomy for intramedullary spinal cord glioblastoma with a 12-year survival. Surg Neurol Int. 2014 Jun 25;5:101. PMID: 25101196
76. Walbert T, Puduvalli V. Primary spinal cord glioblastoma: response to therapy with bevacizumab. Neuro-Oncology journals Dept, Nov 2010; 44-44
77. [Warade AG](http://www.ncbi.nlm.nih.gov/pubmed/?term=Warade%20AG%5BAuthor%5D&cauthor=true&cauthor_uid=24842321), [Misra BK](http://www.ncbi.nlm.nih.gov/pubmed/?term=Misra%20BK%5BAuthor%5D&cauthor=true&cauthor_uid=24842321). Dorsally exophytic cervicomedullary glioblastoma. J Clin Neurosci. 2014 Oct;21(10):1823-4. PMID: 24842321
78. Werner A, Wildi H, Tchicaloff M. Sur **un** **cas** **de** glioblastome multiforme **medullaire** survenant plus **de** deux ans apres excision d’une meme tumeur dans **un** lobe occipital. Schw. Arch. Neurol. 78 (1956), 334–341
79. [Williams SR](http://www.ncbi.nlm.nih.gov/pubmed/?term=Williams%20SR%5BAuthor%5D&cauthor=true&cauthor_uid=21325257), [Parker JR](http://www.ncbi.nlm.nih.gov/pubmed/?term=Parker%20JR%5BAuthor%5D&cauthor=true&cauthor_uid=21325257), [Martin A](http://www.ncbi.nlm.nih.gov/pubmed/?term=Martin%20A%5BAuthor%5D&cauthor=true&cauthor_uid=21325257), [Vitaz T](http://www.ncbi.nlm.nih.gov/pubmed/?term=Vitaz%20T%5BAuthor%5D&cauthor=true&cauthor_uid=21325257), [Parker JC Jr](http://www.ncbi.nlm.nih.gov/pubmed/?term=Parker%20JC%20Jr%5BAuthor%5D&cauthor=true&cauthor_uid=21325257). Plasma cell neoplasm in conjunction with glioblastoma of the conus medullaris. Ann Clin Lab Sci. 2011 Fall;41(1):61-5. PMID: 21325257
80. [Yagi T](http://www.ncbi.nlm.nih.gov/pubmed/?term=Yagi%20T%5BAuthor%5D&cauthor=true&cauthor_uid=9442220), [Ohata K](http://www.ncbi.nlm.nih.gov/pubmed/?term=Ohata%20K%5BAuthor%5D&cauthor=true&cauthor_uid=9442220), [Haque M](http://www.ncbi.nlm.nih.gov/pubmed/?term=Haque%20M%5BAuthor%5D&cauthor=true&cauthor_uid=9442220), [Hakuba A](http://www.ncbi.nlm.nih.gov/pubmed/?term=Hakuba%20A%5BAuthor%5D&cauthor=true&cauthor_uid=9442220). Intramedullary spinal cord tumour associated with neurofibromatosis type 1. Acta Neurochir (Wien). 1997;139(11):1055-60. PMID: 9442220
81. [Yeung YF](http://www.ncbi.nlm.nih.gov/pubmed/?term=Yeung%20YF%5BAuthor%5D&cauthor=true&cauthor_uid=16464801), [Wong GK](http://www.ncbi.nlm.nih.gov/pubmed/?term=Wong%20GK%5BAuthor%5D&cauthor=true&cauthor_uid=16464801), [Zhu XL](http://www.ncbi.nlm.nih.gov/pubmed/?term=Zhu%20XL%5BAuthor%5D&cauthor=true&cauthor_uid=16464801), [Ma BB](http://www.ncbi.nlm.nih.gov/pubmed/?term=Ma%20BB%5BAuthor%5D&cauthor=true&cauthor_uid=16464801), [Hk NG](http://www.ncbi.nlm.nih.gov/pubmed/?term=Hk%20NG%5BAuthor%5D&cauthor=true&cauthor_uid=16464801), [Poon WS](http://www.ncbi.nlm.nih.gov/pubmed/?term=Poon%20WS%5BAuthor%5D&cauthor=true&cauthor_uid=16464801). Radiation-induced spinal glioblastoma multiforme.Acta Oncol. 2006;45(1):87-90. PMID: 16464801
82. [Yoshikawa A](http://www.ncbi.nlm.nih.gov/pubmed/?term=Yoshikawa%20A%5BAuthor%5D&cauthor=true&cauthor_uid=23053494), [Nakada M](http://www.ncbi.nlm.nih.gov/pubmed/?term=Nakada%20M%5BAuthor%5D&cauthor=true&cauthor_uid=23053494), [Watanabe T](http://www.ncbi.nlm.nih.gov/pubmed/?term=Watanabe%20T%5BAuthor%5D&cauthor=true&cauthor_uid=23053494), [Hayashi Y](http://www.ncbi.nlm.nih.gov/pubmed/?term=Hayashi%20Y%5BAuthor%5D&cauthor=true&cauthor_uid=23053494), [Sabit H](http://www.ncbi.nlm.nih.gov/pubmed/?term=Sabit%20H%5BAuthor%5D&cauthor=true&cauthor_uid=23053494), [Kato Y](http://www.ncbi.nlm.nih.gov/pubmed/?term=Kato%20Y%5BAuthor%5D&cauthor=true&cauthor_uid=23053494), [Suzuki S](http://www.ncbi.nlm.nih.gov/pubmed/?term=Suzuki%20S%5BAuthor%5D&cauthor=true&cauthor_uid=23053494), [Ooi A](http://www.ncbi.nlm.nih.gov/pubmed/?term=Ooi%20A%5BAuthor%5D&cauthor=true&cauthor_uid=23053494), [Sato H](http://www.ncbi.nlm.nih.gov/pubmed/?term=Sato%20H%5BAuthor%5D&cauthor=true&cauthor_uid=23053494), [Hamada J](http://www.ncbi.nlm.nih.gov/pubmed/?term=Hamada%20J%5BAuthor%5D&cauthor=true&cauthor_uid=23053494). Progressive adult primary glioblastoma in the medulla oblongata with an unmethylated MGMT promoter and without an IDH mutation. Brain Tumor Pathol. 2013 Jul;30(3):175-9. PMID: 23053494
